# SARS-CoV-2 infects epithelial cells of the blood-cerebrospinal fluid barrier rather than endothelial cells or pericytes of the blood-brain barrier

**DOI:** 10.1186/s12987-023-00479-4

**Published:** 2023-10-24

**Authors:** Chiara Stüdle, Hideaki Nishihara, Sven Wischnewski, Laila Kulsvehagen, Sylvain Perriot, Hiroshi Ishikawa, Horst Schroten, Stephan Frank, Nikolaus Deigendesch, Renaud Du Pasquier, Lucas Schirmer, Anne-Katrin Pröbstel, Britta Engelhardt

**Affiliations:** 1 Theodor Kocher Institute, University of Bern, Bern, Switzerland; 2 Department of Neurotherapeutics, Yamaguchi University, Yamaguchi, Japan; 3 Department of Neurology, Medical Faculty Mannheim, Heidelberg University, Mannheim, Germany; 4 Departments of Neurology, Biomedicine and Clinical Research, Research Center for Clinical Neuroimmunology and Neuroscience Basel (RC2NB), University Hospital Basel and University of Basel, Basel, Switzerland; 5 Laboratory of Neuroimmunology, Neuroscience Research Centre, Department of Clinical Neurosciences, Lausanne University Hospital (CHUV) and University of Lausanne, Lausanne, Switzerland; 6 Laboratory of Clinical Regenerative Medicine, Department of Neurosurgery, University of Tsukuba, Tsukuba, 305-8575 Ibaraki Japan; 7 Pediatric Infectious Diseases, Department of Pediatrics, Medical Faculty Mannheim, Heidelberg University, Mannheim, Germany; 8 Pathology, Institute of Medical Genetics and Pathology, University Hospital Basel and University of Basel, Basel, Switzerland; 9 Service of Neurology, Department of Clinical Neurosciences, Lausanne University Hospital (CHUV), University of Lausanne, Lausanne, Switzerland; 10 Center for Translational Neuroscience and Institute for Innate Immunoscience, Medical Faculty Mannheim, Heidelberg University, Mannheim, Germany; 11 Interdisciplinary Center for Neurosciences, Heidelberg University, Heidelberg, Germany

**Keywords:** SARS-CoV-2, Blood-brain barrier, Blood-cerebrospinal fluid barrier, hiPSC-derived brain microvascular endothelial cells, Choroid plexus epithelial cells

## Abstract

**Background:**

As a consequence of SARS-CoV-2 infection various neurocognitive and neuropsychiatric symptoms can appear, which may persist for several months post infection. However, cell type-specific routes of brain infection and underlying mechanisms resulting in neuroglial dysfunction are not well understood.

**Methods:**

Here, we investigated the susceptibility of cells constituting the blood-brain barrier (BBB) and the blood-cerebrospinal fluid barrier (BCSFB) of the choroid plexus (ChP) to SARS-CoV-2 infection using human induced pluripotent stem cell (hiPSC)-derived cellular models and a ChP papilloma-derived epithelial cell line as well as ChP tissue from COVID-19 patients, respectively.

**Results:**

We noted a differential infectibility of hiPSC-derived brain microvascular endothelial cells (BMECs) depending on the differentiation method. Extended endothelial culture method (EECM)-BMECs characterized by a complete set of endothelial markers, good barrier properties and a mature immune phenotype were refractory to SARS-CoV-2 infection and did not exhibit an activated phenotype after prolonged SARS-CoV-2 inoculation. In contrast, defined medium method (DMM)-BMECs, characterized by a mixed endothelial and epithelial phenotype and excellent barrier properties were productively infected by SARS-CoV-2 in an ACE2-dependent manner. hiPSC-derived brain pericyte-like cells (BPLCs) lacking ACE2 expression were not susceptible to SARS-CoV-2 infection. Furthermore, the human choroid plexus papilloma-derived epithelial cell line HIBCPP, modeling the BCSFB was productively infected by SARS-CoV-2 preferentially from the basolateral side, facing the blood compartment. Assessment of ChP tissue from COVID-19 patients by RNA in situ hybridization revealed SARS-CoV-2 transcripts in ChP epithelial and ChP stromal cells.

**Conclusions:**

Our study shows that the BCSFB of the ChP rather than the BBB is susceptible to direct SARS-CoV-2 infection. Thus, neuropsychiatric symptoms because of COVID-19 may rather be associated with dysfunction of the BCSFB than the BBB. Future studies should consider a role of the ChP in underlying neuropsychiatric symptoms following SARS-CoV-2 infection.

**Supplementary Information:**

The online version contains supplementary material available at 10.1186/s12987-023-00479-4.

## Background

Severe acute respiratory syndrome corona virus 2 (SARS-CoV-2) is the causing agent of corona virus disease 2019 (COVID-19). In addition to respiratory symptoms SARS-CoV-2 can give rise to a multitude of non-respiratory manifestations including such affecting the central nervous system (CNS) [1, 2] or the vasculature [3]. In a subset of patients these manifestations persist for several months after acute infection [4–7]. Pathological mechanisms underlying neuropsychiatric symptoms in COVID-19 have remained largely unknown. CNS-related symptoms can be a result of direct infection of CNS-resident cells by SARS-CoV-2, secondary effects stemming from SARS-CoV-2-induced immune responses or vascular damage, which itself can arise due to direct infection of vascular cells or as a consequence of immune-mediated effects. In brains of deceased COVID-19 patients SARS-CoV-2 RNA was only occasionally detected at low levels and did not show any correlation to neuroinflammatory signatures indicative of virus replication within the CNS [8–11]. In rare cases, SARS-CoV-2 RNA was found in cerebrospinal fluid of living COVID-19 patients [1, 12–14]. Although confounding effects due to the interval between infection and sample acquisition, and lack of sequential sample collection cannot be excluded, these observations do not support significant neurotropism and replication of SARS-CoV-2 within the CNS. Findings from SARS-CoV-2 inoculations of human pluripotent stem cell-derived cerebral organoid cultures that showed no or non-productive infection of neuronal or glial cells [15–21] further corroborate the limited neurotropism and replication of SARS-CoV-2 in the CNS. Moreover, in animal models, which are naturally permissive to the commonly circulating SARS-CoV-2 variants including golden Syrian hamster [22] and non-human primates [23], despite observations of CNS pathology, SARS-CoV-2 transcripts and antigens were not detected in the CNS. Interestingly, in those human post-mortem brain samples, in which SARS-CoV-2 protein was detected, it was often co-localized with blood vessels [10, 17, 24–28]. Reports of increased levels of markers of endothelial activation and injury in the circulation of COVID-19 patients including von Willebrand factor (vWF), vascular cell adhesion molecule 1 (VCAM-1), E-Selectin, glycocalyx degradation products and circulating endothelial cells [29–34], as well as high incidence of thrombotic manifestations in multiple organs of COVID-19 patients [35] raised the question of whether endothelial cells are susceptible to SARS-CoV-2 infection.

In the CNS parenchyma, the microvasculature is lined by highly specialized brain microvascular endothelial cells (BMECs) that form a physical and metabolic barrier. Together with pericytes and astrocyte end feet, BMECs constitute the blood-brain barrier (BBB) contributing to CNS homeostasis by selectively regulating the passage of blood-borne soluble and cellular components [36]. The circumventricular organs and the choroid plexus (ChP) are brain structures that lack a vascular BBB. Instead, their capillaries are fenestrated allowing the free passage of large molecules up to 800 kDa, which is needed for neurosecretory and neurosensory functions [37]. The ChP produces the cerebrospinal fluid (CSF). The ChP stroma is ensheathed by a single-layered epithelium that forms the blood-cerebrospinal fluid barrier (BCSFB) [38]. ChP epithelial cells are connected by tight junctions and face at their basolateral side the vascularized ChP stroma and at the apical side the ventricular space.

Neurological manifestations in COVID-19 could arise due to a compromised BBB or BCSFB [39, 40]. Thus, SARS-CoV-2 infection of BMECs or ChP epithelial cells leading to disruption of their barrier properties may be one of the main reasons that eventually can trigger and impact neuroglial dysfunction as a cause of neuropsychiatric symptoms in COVID-19. Indeed, increased albumin concentration in CSF was detected in up to 50% of individuals affected by COVID-19 when compared to non-infected controls [12–14], and several autopsy studies found increased fibrinogen leakage in different brain regions of COVID-19 patients compared to SARS-CoV-2 naïve patients [24, 26, 41]. Both findings are indicative of large molecule extravasation into the CNS through an altered BBB or BCSFB in COVID-19 patients.

To investigate alterations of the brain barrier in COVID-19 patients, here we assessed the susceptibility of human induced pluripotent stem cell (hiPSC)-derived BMECs [42–44] and hiPSC-derived brain-like pericytes [45, 46] as well as the human ChP papilloma-derived epithelial cell line HIBCPP [47] to SARS-CoV-2 infection. We show that human ChP epithelial cells rather than human BMECs and pericytes are a likely direct target of SARS-CoV-2 infection.

## Methods

### Cell lines

Calu-3 cells derived from the pleural effusion of a 25 years old patient with a lung adenocarcinoma [48] and VeroE6 cells, immortalized African green kidney epithelial cells [49], (both provided by Prof. Robert Rieben, Department of Biomedical Research (DBMR), University of Bern) were grown in Dulbecco’s modified Eagle Medium (DMEM)–GlutaMAX™, 10% (v/v) heat-inactivated fetal bovine serum (FBS), 100 mg/mL streptomycin, 100 IU/mL penicillin, 1% (w/v) non-essential amino acids and 15 mM HEPES (all Gibco). HIBCPP (a cell line obtained from a choroid plexus papilloma [47]) were maintained in DMEM/F12, 10% v/v heat-inactivated FBS (Gibco), 5 µg/mL human insulin (Sigma Aldrich), 4mM L-Glutamine (Gibco) and 100 mg/mL streptomycin, 100 IU/mL penicillin (HIBCPP-10 medium). Human induced pluripotent stem cells (hiPSCs) were generated from erythroblasts reprogrammed through nucleofection with plasmids encoding for OCT4, shRNAp53, SOX2, KLF4, L-Myc, and Lin28 [50]. In this study 2 iPSC clones per donor were used: LNISi001-A/B, LNISi002-A/B, LNISi003-A/B, LNISi004-A/B, with the following age/sex: 30/female, 50/male, 49/female, 33/female. HiPSCs were maintained on growth factor reduced Matrigel^®^ (Corning) or growth factor reduced and stem cell certified Cultrex™ (R&D) coated plates in mTeSR^TM^1 medium (STEMCELL Technologies) [42, 51].

### Differentiation of hiPSCs to extended endothelial culture method (EECM)-BMECs

Extended endothelial culture method (EECM) brain microvascular endothelial cell (BMEC)-like cells (for simplicity reasons referred to EECM-BMECs throughout the manuscript) were differentiated from hiPSC via a mesoderm intermediate by canonical WNT pathway induction using small molecule CHIR99021, and a CD31^+^34^+^ endothelial progenitor cell stage to BMECs exhibiting barrier properties and a mature immune phenotype exactly as described before [42, 43, 52]. In brief, hiPSCs were seeded at a density of 23’000–51’000 cells/cm^2^ depending on the donor on 12-well plates coated with growth factor reduced Matrigel^®^ (Corning) in mTESR^TM^1 (STEMCELL Technologies) medium supplemented with 5 µM ROCK inhibitor Y-27,632 (Tocris). The next 2 days mTeSR^TM^1 without ROCK inhibitor was used and on days 0 and 1 of differentiation, the medium was changed to basal LaSR1 medium (Advanced DMEM/F12, 2.5 mM GlutaMAX™ (Gibco), 60 ng/mL ascorbic acid (Sigma Aldrich)) supplemented with 8 µM CHIR99021 (Selleckchem). During the 3 following days, the medium was changed to basal LaSR1 without CHIR99021. On day 5, CD31^+^ endothelial progenitor cells (EPCs) were purified by magnetic-activated cell sorting using a FITC-conjugated anti-human CD31 antibody (Miltenyi Biotec, clone AC128) and EasySep Human FITC Positive Selection Kit II (STEMCELL Technologies) with an EasySep Magnet kit (STEMCELL Technologies). EPCs were seeded at a density of 10’000–20’000 cells/cm^2^ on 6-well plates coated with 10 ug/mL collagen type IV (ColIV) from human placenta (Sigma Aldrich) in hESCR1 medium (hESFM (Gibco), 2% v/v B27 supplement (STEMCELL Technologies), 20 ng/mL FGF-2 (R&D)) supplemented with 5 µM ROCK inhibitor. 24 h later, the Rock inhibitor was removed and cells were cultured until endothelial cell colonies started touching smooth muscle-like cell (SMLC) colonies with medium changes every other day. For selective passage to separate endothelial cells from SMLCs, cell monolayers were treated with Accutase^®^ (Sigma Aldrich) while being observed under the microscope. As soon as the endothelial cells acquired a round morphology, they were dislodged by gentle tapping, while SMLCs still remained adherent. EECM-BMECs were seeded again at 10’000–20’000 cells/cm^2^ and selective passage was repeated until no SMLCs remained in the culture. EECM-BMECs were used for expression and infection experiments at passages 4 to 6. For transendothelial/-epithelial electrical resistance measurements, EECM-BMECs were seeded on Transwell^®^ filter inserts (1.12 cm^2^, 0.4 μm pore size, polycarbonate, (Costar)) coated with 400 ug/mL ColIV and 100 ug/mL bovine fibronectin (Sigma Aldrich) and cultured in the cellZscope2 (nanoanalytics) for 6 days.

### Differentiation of hiPSCs to defined medium method (DMM)-BMECs

Differentiation of BMECs by the defined medium method (DMM), which induces iPSCs via a mesoderm stage by canonical WNT pathway activation using CHIR99021 to VEGFR2^+^ endothelial progenitors that are further specified to barrier forming BMECs by endothelial medium supplemented with retinoic acid (RA), was performed according to the original protocol [42, 44]. Briefly, hiPSCs were seeded at 37’000–42’000 cells/cm^2^ in Matrigel^®^ or Cultrex™ coated 6-well plates in mTeSR^TM^1 medium supplemented with 10 µM ROCK inhibitor. On the following 2 days medium was changed to mTeSR^TM^1 without ROCK inhibitor. On day 0, medium was changed to DeSR1 medium (DMEM/F12, 1% (w/v) nonessential amino acids (Gibco), 1 mM GlutaMAX™, and 0.1 mM β-mercaptoethanol (Sigma Aldrich)) supplemented with 6 µM CHIR99021 and on days 1 to 5 DeSR2 (DeSR1 supplemented with 2% B27) was used. On day 6, medium was changed to hESCR1 supplemented with 10 μm RA and on day 8, cells were dislodged using Accutase^®^ and seeded at 0.6-1 million cells/cm^2^ in hESCR1 plus RA in culture vessels coated with Cultrex™. 1 day later medium was changed to hESFM supplemented with 2% B27 only (hESCR2). DMM-BMECs at differentiation day 11–12 were used for expression and infection experiments.

### Differentiation of hiPSCs to brain pericyte-like cells (BPLCs)

Brain pericyte-like cells (BPLCs) were differentiated from hiPSCs via neural crest stem cells (NCSCs) as previously described [46]. In brief, hiPSCs were seeded at a density of 92’000 cells/cm^2^ in Matrigel^®^ coated 6-well plates in mTeSR^TM^1 medium supplemented with 10 µM ROCK inhibitor (day − 1). The next day medium was changed to E6-CSFD medium (TeSR^TM^-E6 medium (STEMCELLTechnologies), 10 µM CHIR99021, 10 µM SB431542 (Tocris), 100 ng/mL FGF-2, 1 µM dorsomorphin (Tocris), 22.5 µg/mL heparin (Sigma Aldrich)). The medium was refreshed every day until day 15 of differentiation. In between when the cells reached full confluence, they were passaged 1:6 into new 6-well plates. On day 15, p75 neurotrophin receptor (p75NTR / CD271) positive NCSCs were purified by magnetic-activated cell sorting using Neural Crest Stem Cell MicroBeads (Miltenyi Biotec) and LS columns (Miltenyi Biotec). Purified NCSCs were seeded at a density of 10,000 cells/cm^2^ in uncoated 6-well plates in E6-CSFD medium supplemented with 10 µM ROCK inhibitor. The next day, medium was changed to TeSR^TM^-E6 supplemented with 10% v/v FBS (Gibco) to initiate pericyte differentiation. The medium was refreshed every day and cells were passaged 1:2 after reaching 100% confluence. From day 22 onwards the cells were considered as BPLCs. BPLCs between day 22–30 were used for expression and infection experiments.

### Generation of SARS-CoV-2 stock and handling of SARS-CoV-2 infected cultures

Calu-3 cells were inoculated with SARS-CoV-2/München-1.1/2020/929 bearing D614G mutation compared to the original Wuhan strain (provided by PD Dr. Ronald Djikman, Institute for Infectious Diseases (IFIK), University of Bern) at multiplicity of infection (MOI) of 0.01 and supernatant was collected by centrifugation 3 days post infection. The integrity of the virus genome was verified by sequencing and the virus titer was determined by the medium tissue culture infectious dose (TCID_50_) assay on Vero-E6 cells. All SARS-CoV-2 related work was performed at biosafety level 3 (BSL3) in a laboratory at SITEM Insel operated by IFIK of the University of Bern. For further analysis outside of the BSL3 laboratory, all culture vessels were fixed for at least 30 min in 10% buffered formalin solution (formafix) to inactivate SARS-CoV-2.

### Cellular Infection with SARS-CoV-2

For infection with SARS-CoV-2, EECM-BMEC at passages 3–5 were seeded at 90’000 cells/cm^2^ in a chamber slide (0.56 cm^2^ culture area) with µ-membrane (ibidi) coated with 400 µg/mL ColIV from human placenta and 100 µg/mL bovine fibronectin. When the cells reached a confluent monolayer, SARS-CoV-2 was diluted in hESCR1 to 5’000 or 50’000 TCID_50_/well (corresponding to a MOI of 0.09–0.9) and inoculated for 1-1.5 h, cells were washed 3x with PBS and cultured for another 3 days. After washing and 24, 48 and 72 h post infection (hpi) half of the medium volume was collected and stored at -80 °C till usage for virus titer determination by TCID_50_ assay, and replenished with fresh medium. 72 hpi, chamber slides were fixed for subsequent immunofluorescence (IF) staining. For prolonged incubation with SARS-CoV-2 the inoculum was prepared with SMLC conditioned medium and was incubated for 24 h prior to washing and fixation. For stimulation with pro-inflammatory cytokines, TNFα and IFNγ (both R&D) were added at 1 ng/mL and 20 IU/mL, respectively, in SMLC conditioned medium to EECM-BMECs 16–20 h prior to SARS-CoV-2 infection.

BPLCs were used at days 22–29 of differentiation and seeded at 10’000–80’000 cells/cm^2^ in a chamber slide with µ-membrane coated with 100 ug/mL bovine fibronectin and 1–2 days later, BPLCs were inoculated for 1-1.5 h with SARS-CoV-2 diluted to 1’000-5000 TCID_50_/ well (corresponding to a MOI of 0.03–0.17) as low inoculum and 10’000–50’000 TCID_50_/ well (corresponding to a MOI of 0.3–1.7) as high inoculum in E6 medium with 10% FBS, washed 3 times with PBS and cultured for another 72 h before fixation. Samples for the TCID_50_ assay were collected as described above.

Calu-3 cells were used from passage 28 to 50 and were seeded at 55’000 cells/cm^2^ in a chamber slide with µ-membrane (ibidi) coated with 100 ug/mL bovine fibronectin and 4 days later were inoculated for 1-1.5 h with SARS-CoV-2 diluted to 1000 TCID_50_/ well (corresponding to a MOI of 0.02) in Calu-3 medium, washed 3 times with PBS and cultured for another 72 h prior to fixation. Samples for the TCID_50_ assay were collected as described above.

Vero-E6 cells were used from passage 30 to 50 and were seeded at 70’000 cells/cm^2^ in a chamber slide with µ-membrane (ibidi) coated with 100 ug/mL bovine fibronectin and 1 day later were inoculated for 1-1.5 h with SARS-CoV-2 diluted to 400 TCID_50_/ well (corresponding to a MOI of 0.007) in Calu-3 medium, washed 3 times with PBS and cultured for another 72 h prior to fixation. Samples for the TCID_50_ assay were collected as described above.

DMM-BMECs at day 8 of differentiation were seeded on Matrigel^TM^-coated Transwell^®^ filter inserts (0.33 cm^2^, 0.4 μm pore size, polycarbonate, (Costar)) and cultured in the cellZscope2 (nanoanalytics) for TEER determination. The replicates that showed the highest TEER values were used for infection experiments. DMM-BMECs at day 10–11 of differentiation were inoculated from the apical side with SARS-CoV-2 stock diluted in hESCR2 medium to 10’000 or 100’000 TCID_50_/ filter insert (corresponding to a MOI of 0.02 or 0.2) for 1–1. 5 h, 3x washed with PBS and replenished with fresh medium. After washing, 24, 48 and 72 hpi an aliquot of medium from the top (apical) compartment was collected, replenished and stored at -80 °C till usage for viral titer determination by TCID_50_ assay. Filter membranes at 24 hpi and 72 hpi were fixed for subsequent analysis by IF staining.

HIBCPP were used between passages 22–32 and were seeded on inverted filter inserts (0.33 cm^2^, 5 μm pore size, PET, (Millipore or Sarstedt)) at 100’000 cells/ filter in HIBCPP-10 medium as described previously [53]. Filter inserts were brought back to a hanging position 24 h later (day 1) and were transferred to the cellZscope2 for TEER determination. When a TEER of approximately 100 Ωcm^2^ was reached (usually at day 3–4) the medium was changed to medium containing only 1% FBS (HIBCPP-1). On day 5 or when a TEER of approximately 300–500 Ωcm^2^ was reached, HIBCPP cells were inoculated with the same volume of medium from either the basolateral or the apical side with SARS-CoV-2 diluted in HIBCPP-1 medium to 100’000 TCID_50_/ filter insert (corresponding to a MOI of 0.7) for 1.5 h. Filter inserts were washed 3x with PBS and replenished with fresh HIBCPP-1 medium. The further procedure was the same as described above for DMM-BMECs, except that medium from the top (basolateral) and bottom (apical) compartment was collected and replenished in 24 h time intervals.

For antibody mediated blocking of ACE2 on DMM-BMEC and HIBCPP, SARS-CoV-2 and anti-ACE2 blocking antibody (10 µg/mL, adipogen, AG-20 A-0037PF-C10) or mouse IgG1 isotype control (10 ug/mL, R&D) were co-incubated for 1-1.5 h on the apical side of DMM-BMECs and on the basolateral side of HIBCPP. After washing and medium replenishment DMM-BMECs and HIBCPP were cultured for 48 and 72 h, respectively, prior to fixation and IF staining.

### Tissue culture Infection dose 50 (TCID_50_) assay

VeroE6 cells were seeded at 20–25’000 cells / well in a 96-well plate and the following day, the cell supernatant previously collected from infected cell cultures at different time points post-infection as described above was added in a 10-fold dilution series in quadruplicates. 72 h to 96 hpi, plates were fixed and stained with crystal violet. Wells with cytopathogenic effects were counted and TCID_50_/mL was calculated using the Spearmann-Kärber method [54]. The TCID_50_ value indicates the virus dose when half of the cells undergo cytopathogenic effects. To convert to MOI, TCID_50_ values were multiplied with 0.69 to get an estimate of plaque forming units [55] and divided by the number of initially seeded cells.

### Immunofluorescence staining and image quantification

Cells cultured on filter membranes or chamber slides were washed 3x in PBS after fixation, blocked and permeabilized in blocking buffer (5% skimmed milk, 0.3% TritonX in Tris buffered saline (TBS). Primary antibodies were incubated in blocking buffer followed by incubation of fluorophore-conjugated secondary antibodies (Life technologies or Jackson Immunology Research), DAPI and phalloidin-TRITC (Life technologies) in blocking buffer and mounting in Mowiol (Sigma Aldrich). The following primary antibodies were used after fixation at room temperature with 4% paraformaldehyde (PFA) for 10–15 min or in case the samples were handled at BSL3 in buffered formalin for at least 30 min: rabbit-anti-SARS-CoV-nucleocapsid protein (NP) (1:1000, Rockland antibodies, 200-401-A50), mouse-anti-VE-Cadherin (1:200, Santa Cruz, sc-9989), mouse-anti-E-Cadherin (1:300, BD biosciences, 610,181), mouse-anti-NG2 (1:100, Millipore, MAB1229), rabbit-anti-PDGFRb (1:100, Cell Signaling Technology, 28E1), mouse-anti-calponin (1:3000, Sigma Aldrich, C2687), rabbit-anti-SM22 (1:1000, abcam, ab14106), mouse-anti-SMA conjugated to Cy3 (1:100, Sigma Aldrich, C6198), goat-anti-ACE2 (1:100, R&D, AF933) and after fixation in methanol at -20 °C: mouse-anti-claudin-5 (1:200, Invitrogen, 35-2500), mouse-anti-occludin (1:100, Invitrogen, 33-1500), mouse-anti-claudin-3 (1:100, Life technologies, 34-1700). For cell surface staining of ACE2 (1:100, adipogen, AG-20 A-0037PF-C10), TMPRSS2 (1:50, abcam, ab280567), ICAM-1 (1:100, Biolegend, 353,102) and VCAM-1 (1:100, BD Biosciences, 555,645) live cells were stained in medium at 37 °C and subsequently fixed with 4% PFA for 10–15 min or in case the samples were handled at BSL3 in buffered formalin for at least 30 min. Images were acquired using a LSM 800 confocal microscope (Zeiss) with a 25x water or a 40x oil objective and Nikon widefield microscope with a 40x objective. For quantification of NP^+^ DMM-BMEC or HIBCPP, tile stack images of the entire filter membrane were acquired at an Axio Observer widefield microscope (Zeiss) using a 10x objective. NP^+^ areas of DMM-BMEC and HIBCPP monolayers were quantified in ImageJ using the threshold tool and normalized to the area covered by DAPI positive nuclei.

### qRT-PCR

For RNA isolation, cells were grown in well plates up to 100% confluence. RNA was isolated using the High pure RNA Isolation kit (Roche) according to the manufacturer’s protocol. RNA quantity was determined using a nanodrop and 300 ng total RNA were reverse transcribed with Maxima H reverse transcriptase and oligo dT primer (both Thermo Fisher Scientific). qRT-PCR was performed using Takyon™ Rox SYBR® MasterMix dTTP Blue (Eurogenetec) on a Viia7 RTqPCR machine. The geometric mean of the Ct-value of β-actin and GAPDH served as the reference to determine ΔCt-values. The used primers are listed in Table 1.

**Table 1:** Used primers for qRT-PCR

*ACE2* forward	TCCATTGGTCTTCTGTCACCCG
*ACE2* reverse	AGACCATCCACCTCCACTTCTC
*TMPRSS2* forward	CCTCTAACTGGTGTGATGGCGT
*TMPRSS2* reverse	TGCCAGGACTTCCTCTGAGATG
*BSG* forward	GGCTGTGAAGTCGTCAGAACAC
*BSG* reverse	ACCTGCTCTCGGAGCCGTTCA
*NRP1* forward	AACAACGGCTCGGACTGGAAGA
*NRP1* reverse	GGTAGATCCTGATGAATCGCGTG
*ACTB* forward	CACCATTGGCAATGAGCGGTTC
*ACTB* reverse	AGGTCTTTGCGGATGTCCACGT
*GAPDH* forward	GTCTCCTCTGACTTCAACAGCG
*GAPDH* reverse	ACCACCCTGTTGCTGTAGCCAA

### Western blotting

For protein isolation, cells were grown in well plates up to 100% confluence. Cells were lysed in RIPA buffer supplemented with protease inhibitors (cOmplete EDTA-free, Roche). Protein quantification was done using a Pierce BCA assay kit (Thermo Fisher Scientific) following the manufacturer’s manual. 25 ug protein per lane (or as stated in the figure legend) were resolved by 8% SDS-PAGE and transferred to a nitrocellulose membrane (Amersham) using a semi-dry transfer cell (BioRad). Membranes were blocked in Rockland blocking buffer (Rockland), incubated with primary antibodies mouse-anti-β-actin (1:2000, Sigma Aldrich, A5316), goat-anti-ACE2 (1:200, R&D, AF933) or rabbit-anti-ACE2 (1:500, abcam, ab15348) and subsequently with IRDye-conjugated secondary antibodies (life technologies) in 5% BSA in TBS containing 0.1% Tween20. Membranes were imaged using an Odyssey IR reader (LI-COR). Quantification was done using Image Studio Lite (LI-COR). The background corrected signal of the ACE2 band at approximately 130 kDa was normalized to the signal of the β-actin band and subsequently to the relative band intensity of a Calu-3 cell control sample.

To study the effect of type I interferon signalling on ACE2 expression in EECM-BMECs, recombinant IFN-α (R&D) at 1 or 0.1 ng/mL was supplemented in hESCR1 medium or SMLC-conditioned medium for 20 h prior to cell lysis.

### Flow cytometry

For flow cytometry, cells were grown in 24-well plates and detached using Accutase^®^ in the case of EECM-, DMM-BMECs and BPLCs and detached using trypsin in the case of Calu-3 and HIBCPP. Staining with primary antibodies conjugated to fluorophores was performed at 4 °C in FACS buffer (PBS, 2% FBS, 0.1% NaN_3_). Samples were acquired using AttuneNxT (Thermo Fisher Scientific). The following primary antibodies were used: anti-NRP1-PE, clone 12C2 (BioLegend); anti-CD147-Alexa 647, clone HIM6 (BioLegend); anti-NG2, clone 9.2.27 (Millipore); anti-PDGRβ, clone 28D4; anti-CD144-PerCpCy5.5, clone 55-7H1; anti-CD31-APCCy7, clone WM59; anti-CD102-PE, clone CBR-IC2/2 (all from BD Bioscience). For calculating the delta mean fluorescent intensity (MFI), the geometric MFI of the isotype control-stained sample was subtracted from the MFI of the stained sample.

### Human brain tissue samples

Autopsy was performed at the Institute of Medical Genetics and Pathology at the University Hospital of Basel, Switzerland. Brains were removed upon opening the skull with a handsaw, avoiding aerosolization of SARS-CoV-2, and *in toto* fixed in 4% (w/v) phosphate-buffered formalin as recently described [56]. The study was approved by the Ethics Committee of Northwestern and Central Switzerland (ID 2020 − 00629 and 2020 − 00969). Formalin-fixed paraffin-embedded (FFPE) tissue was routinely prepared to 4 μm thick sections. Patient information is provided in Table 2. None of the patients had received a SARS-CoV-2 vaccine. Data on choroid plexus (*SARS-CoV-2* and *ACE2*) for P1 was reported before [57] and histological assessment of non-choroid plexus brain regions for all subjects was previously reported by Deigendesch et al. [58].

**Table 2:** Patient information

Patient number	Sex	Age (years)	Days from COVID-19 symptom onset to death	Postmortem delay (hours)	Autoptic cause of death	Pre-existing neurological conditions
P1	F	67	13	20	Respiratory failure	Chronic multiple sclerosis
P2	M	96	unclear	24	Respiratory failure	Parkinson`s disease
P3	M	66	23	11	Respiratory failure	none
P4	M	59	at least 19	39	Respiratory failure	none
C1	M	89	Non-COVID-19 control	7	Cardiac failure with bronchopneumonia	none

### Immunofluorescence staining of human brain tissue samples

FFPE tissue slides were re-hydrated, exposed to heat-mediated antigen retrieval in EDTA buffer, pH 9, blocked and permeabilized in phosphate buffered saline (PBS) with 10% donkey serum and 0.1% triton and stained with goat-anti-ACE2 (1:50, R&D, AF933) and rabbit-anti-vWF (1:300, Dako, A0082) over night at 4 °C in PBS with 2% donkey serum. Following incubation with fluorophore-conjugated secondary antibodies and Dapi, sections were mounted in mowiol. For ACE2 quantification in brain stem sections, vWF^+^ vessels and vWF^+^ with an associated ACE2 signal were manually counted in 15 images of 256 × 256 μm per patient.

### Fluorescent multiplex in situ RNA hybridization of human brain tissue samples

Fluorescent multiplex in situ RNA hybridization was performed on FFPE tissue slides as previously described [57, 58] using the RNAscope Multiplex Assay v2 (ACD Biotechne) with the TSA Plus Fluorophores (Fluorescein, Cyanine 3, Cyanine 5). The following manual RNAscope assay probes were used: Hs-*TTR*, Hs-*ACE2*, Hs-*VWF*, *V-nCoV2019-S*. Images were taken with a THUNDER imaging system (Leica DM6 B microscope) using a 63x lens. All taken images are Z-stack images and were processed using Fiji ImageJ (v2.3.0/1.53f51). *ACE2*^*+*^, *SARS-CoV-2*^*+*^, *TTR*^*+*^ and respective double positive cells were manually counted in 2 images per patient.

### Statistics

The statistical analysis was performed using GraphPad Prism software 9. Data is presented as mean +/-standard error mean. To compare two groups, statistical significance was assessed by unpaired T-test with Welch’s correction, while more groups were analyzed by one- or two-way ANOVA followed by Tukey’s multiple comparison test. The respective statistical methodology used is indicated in the figure legends. Statistical significance was set at P < 0.05 and the respective values are indicated in the graph.

## Results

### In vitro models for the human blood-brain and blood-cerebrospinal fluid barrier

Brain autopsy studies detected SARS-CoV-2 protein in microvessels [10, 24–26, 28], however, it remained unclear, whether brain endothelial cells or vascular mural cells such as pericytes may be infected and whether SARS-CoV-2 can replicate in either of these cell types. As a cellular model for testing the susceptibility of BBB endothelial cells to SARS-CoV-2 infection we used two different models of hiPSC-derived BMECs, namely extended endothelial cell culture (EECM)-BMEC-like cells (here referred to as EECM-BMECs) [42, 43] and defined medium method (DMM)-BMECs [44]. EECM-BMEC and DMM-BMEC monolayers displayed continuous junctional localization of tight junction proteins (Fig. S1A, Additional file 1), but showed a distinct morphology. EECM-BMECs displayed a flat and spindle-shaped morphology typical of endothelial cells and stained positive for vascular endothelial (VE)-cadherin (Fig. S1A, Additional file 1). DMM-BMECs exhibited a cuboidal morphology resembling epithelial cells and showed strong immunoreactivity for epithelial (E)-cadherin (Fig. S1A, Additional file 1). We failed to reproducibly detect the endothelial proteins platelet endothelial cell adhesion molecule (PECAM-1), VE-cadherin and intercellular adhesion molecule 2 (ICAM-2) by flow cytometry in DMM-BMECs (Fig. S1B, Additional file 1). EECM-BMECs but not DMM-BMECs were previously shown to display the complete set of cell adhesion molecules necessary for interaction with immune cells upon stimulation with pro-inflammatory cytokines [42]. In accordance with previous observations DMM-BMECs exhibited significantly higher trans-endothelial/epithelial electrical resistance (TEER) when compared to EECM-BMECs [42, 44] (Fig. S1C, Additional file 1).

To test the susceptibility of brain pericytes to SARS-CoV-2 infection we specifically used brain pericyte-like cells (BPLCs) differentiated from hiPSC via neural crest stem cells (NCSCs), mimicking the developmental origin of human forebrain pericytes [45, 46]. BPLCs stained 100% positive for neural/glial antigen 2 (NG2) and platelet-derived growth factor receptor β (PDGFRβ) as measured by flow cytometry (Fig. S1D, Additional file 1). BPLC cultures also stained positive for the actin-associated proteins calponin and transgelin (SM22), while only very few cells stained positive for smooth muscle actin (SMA) (Fig. S1E, Additional file 1) as expected for pericytes [45, 46].

As SARS-CoV-2 could also reach the BCSFB through the fenestrated capillaries in the ChP stroma we included the human choroid plexus papilloma-derived epithelial cells HIBCPP [47] as a model for the BCSFB in our study. In accordance with previous observations by us and others [59] HIBCPP grown on filter inserts formed a monolayer with mature adherence and tight junctions (Fig. S1F, Additional file 1).

### HIBCPP cells show higher expression of ACE2 and TMPRSS2 than cellular models of the human BBB

**Fig. 1:**
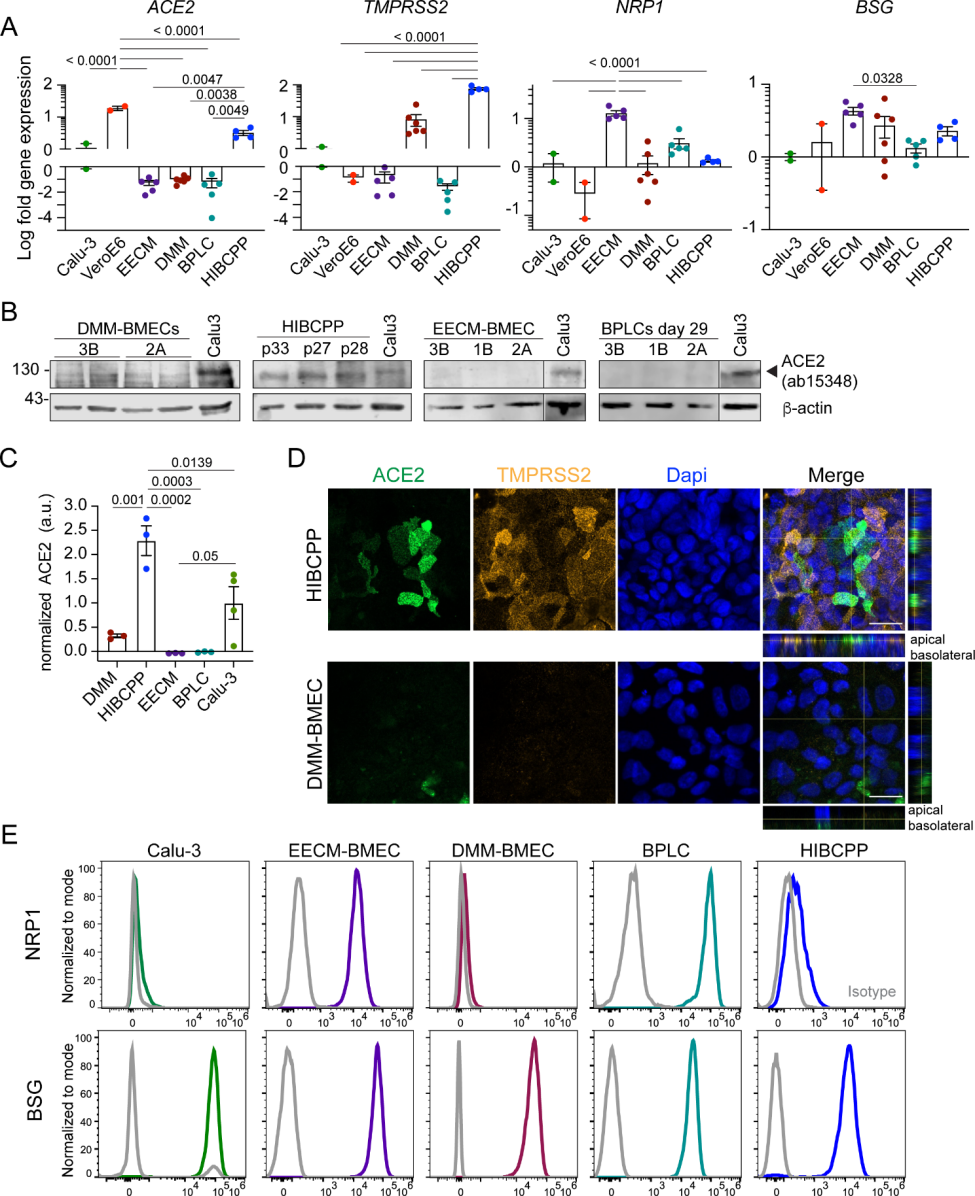
Expression of SARS-CoV-2 host factors in brain barrier cells. **(A)** qRT-PCR analysis of *ACE2*, *TMPRSS2*, *neuropilin 1* (*NRP1*) and *basigin* (*BSG*) as expression fold change relative to Calu-3 cells is shown. *β-actin* and *GAPDH* were used as reference genes. Each dot represents an independent differentiation for EECM-BMECs from 5, DMM-BMECs from 6 and BPLCs from 5 iPSC clones, or an independent replicate of HIBCPP, VeroE6 and Calu3. P-values were determined using one-way Anova followed by Tukey’s test to correct for multiple comparison. **(B)** Western Blot analysis of ACE2 using ab15348 raised against the intracellular domain of ACE2 with β-actin as a loading control is shown. Glycosylated ACE2 is expected at 130 kDa (indicated with arrowhead). Each lane represents an independent differentiation of EECM-BMECs, DMM-BMECs or BPLCs with the clone ID of the iPSC indicated and a replicate of HIBCPP with displayed passage number. 25 µg of protein was loaded per lane. **(C)** Quantification of relative ACE2 expression by Western blotting is shown normalized to the expression in Calu-3 cells. Each dot represents a clone for EECM-BMECs, DMM-BMECs or BPLCs (3 iPSC clones each) and a replicate of HIBCPP cells. P-values were determined by one-way Anova with Tukey’s test to correct for multiple comparison. **(D)** Confocal images from immunofluorescence staining for TMPRSS2 (orange) and ACE2 (green) using AG-20 A-0037PF antibody in DMM-BMEC and HIBCPP cells are shown. Nuclei were stained with DAPI (blue). A single Z-plane and for the merge in addition the orthogonal sections are shown. Scale bar = 20 μm. The images are representative of DMM-BMEC differentiations from 3 clones and 3 replicates of HIBCPP. **(E)** Representative histogram overlays from flow cytometry analysis for NRP1 or BSG on EECM-BMEC (from 4 differentiations, purple), BPLC (from 2 differentiations, petrol), DMM-BMEC (1 differentiation, dark red) and HIBCPP (3 replicates, blue), of Calu-3 (1 replicate, green) with isotype control (grey) are shown

First, we profiled the gene expression of the main known mediators of SARS-CoV-2 cell entry. To this end, we tested mRNA expression of the entry receptor angiotensin converting enzyme 2 (ACE2) and the host protease transmembrane serine proteinase 2 (TMPRSS2) [60, 61]. We also tested expression of neuropilin 1 (NRP1), which was shown to serve as a co-entry receptor in case of low ACE2 expression [62] by binding to the CendR motif that is accessible on furin-cleaved SARS-CoV-2 spike protein domain S1 [63]. Finally, we determined expression of extracellular matrix metalloproteinase inducer EMMPRIN previously described as basigin (BSG) and proposed as an alternative SARS-CoV-2 entry receptor [64]. BSG is of particular interest because it is specifically and highly expressed in brain endothelial cells and ChP epithelial cells [65, 66]. The human lung adenocarcinoma cell line Calu-3 and the African green monkey kidney epithelial cell line VeroE6, which are both highly susceptible to SARS-CoV-2 infection [67] were included as controls. *ACE2* expression was highest in Vero E6 cells compared to all other cell types (Fig. 1A). HIBCPP cells expressed *ACE2* and *TMPRSS2* significantly higher than EECM-BMECs, DMM-BMECs and BPLCs (Fig. 1A). *TMPRSS2* expression was also detected in DMM-BMECs, but not in EECM-BMECs and BPLCs. EECM-BMECs expressed *NRP1* significantly higher than all other cell types and *BSG* was expressed in all the cell types with highest values in EECM-BMECs (Fig. 1A). At the protein level we could detect glycosylated ACE2 protein in HIBCPP and Calu-3 and very faintly in DMM-BMECs cells by Western Blotting using an antibody recognizing an intracellular epitope of ACE2, while ACE2 was absent in EECM-BMECs and BPLCs (Fig. 1B-C). Employing an alternative anti-ACE2 antibody raised against full-length ACE2, a band corresponding to glycosylated ACE2 could be detected in HIBCPP and Calu-3 cells but not in DMM-BMECs (Fig. S2A-B, Additional file 1). Immunofluorescence (IF) staining confirmed TMPRSS2 immunostaining in the majority of HIBCPP cells and ACE2 immunostaining in a subset of HIBCPP cells (Fig. 1D and Fig. S2C, Additional file 1). TMPRSS2 and ACE2 were more prominently localized at the apical surface of HIBCPPs (Fig. 1D and Fig. S2D, Additional file 1). In DMM-BMECs we could not detect ACE2 expression by immunostaining using two different antibodies (Fig. 1D and Fig. S2C, Additional file 1). Neither could we detect a positive signal for TMPRSS2 (Fig. 1D) despite its expression at the mRNA suggesting protein levels to be below the detection limit. While EECM-BMECs and BPLCs stained 100% positive for NRP1 as detected by flow cytometry, NRP1 was almost absent on DMM-BMECs, Calu3 and HIBCPP (Fig. 1E) while 100% of all the cell types stained positive for BSG (Fig. 1E). In summary, these results demonstrate that HIBCPP cells express SARS-CoV-2 host factors as observed in ChP epithelial cells in vivo [68–71]. EECM-BMECs displayed expression of SARS-CoV-2 host factors similarly as BMECs in vivo, while the lack of ACE2 expression in BPLCs was unexpected, since vascular ACE2 expression was rather allocated to pericytes than to endothelial cells in in vivo studies [26, 72–75].

### EECM-BMECs, DMM-BMECs and BPLCs differ in their susceptibility to SARS-CoV-2 Infection

**Fig. 2:**
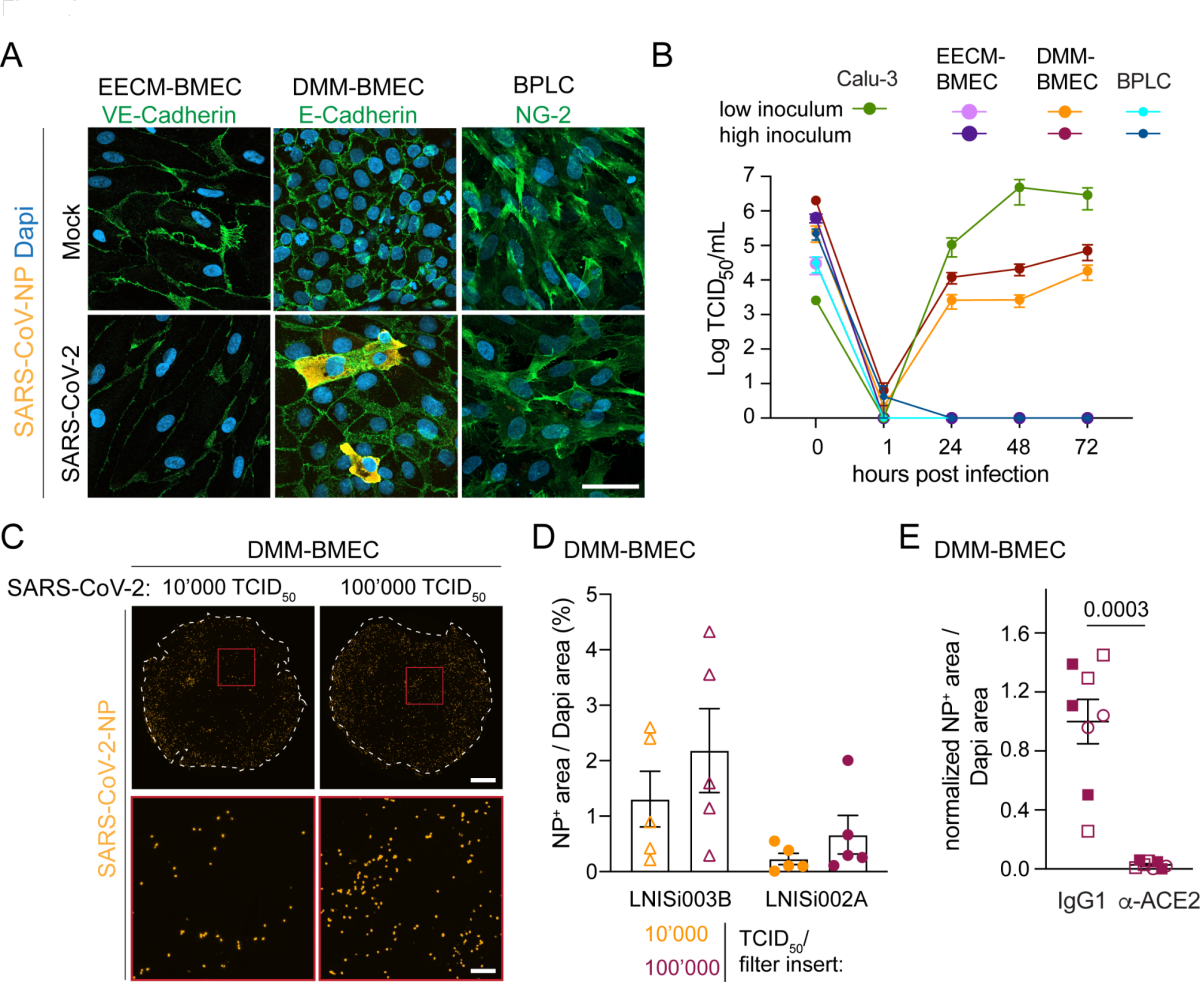
EECM-BMECs, DMM-BMECs and BPLCs differ in their susceptibility to SARS-CoV-2 infection. **(A)** Representative confocal images from immunofluorescence staining of SARS-CoV-nucleocapsid protein (NP) (orange) and VE-cadherin in EECM-BMECs (green), E-cadherin (green) in DMM-BMECs or neural/glial antigen-2 (NG2, green) in BPLCs 72 hpi with SARS-CoV-2 or Mock are shown. Nuclei were stained with DAPI (blue). Scale bar = 50 μm. **(B)** Quantification of released virions into the supernatant 1–72 hpi with SARS-CoV-2 by TCID_50_ assay. Measurement was done in duplicates from a total of 2 experiments with Calu-3 cells, 3 experiments with EECM-BMECs (each time a different iPSC clone and a low and high inoculum concentration), 2 experiments with DMM-BMECs derived from 2 iPSC clones (and a low and high inoculum concentration) and 4 experiments with BPLCs (with in total 5 iPSC clones and a low and high inoculum concentration). **(C)** Representative tile stack images of whole filter inserts of DMM-BMECs 72 hpi with SARS-CoV-2 at 10’000 and 100’000 TCID_50_/filter insert stained for SARS-CoV-NP (orange) are shown. White dashed circle indicates border of the filter inserts, red box delineates inset. Scale bar = 1 mm and for inset 200 μm. **(D)** Quantification of area of SARS-CoV-NP positivity normalized to area covered by DAPI is shown. Each dot represents one replicate from in total 2 experiments with 2 independent DMM-Differentiations from two clones. **(E)** Quantification of area of SARS-CoV-NP positivity normalized to area covered by DAPI is shown 48 hpi with SARS-CoV-2 at 100’000 TCID_50_/filter in presence of an anti-ACE2 blocking antibody or IgG control antibody. Data is normalized to IgG control. Each symbol type indicates an iPSC clone and each symbol is a replicate from in total 3 experiments with 3 DMM-differentiations from 3 donors. P-value was calculated by unpaired T-test with Welch’s correction

To test whether cells composing the BBB can be infected with SARS-CoV-2 in vitro, in EECM-BMECs, BPLCs and DMM-BMECs we determined the presence of SARS-CoV-nucleocapsid protein (NP) by IF staining and measured the release of active virions into the medium by median tissue culture infectious dose (TCID_50_) assays up to 72 h post infection (hpi). A SARS-CoV-NP^+^ signal could only be detected in DMM-BMECs 72 hpi (Fig. 2A) and accordingly, we did not detect active SARS-CoV-2 in the supernatants of EECM-BMECs and BPLCs by TCID_50_ assay confirming that neither EECM-BMECs nor BPLCs were productively infected by SARS-CoV-2 with any of the tested inoculate concentrations (Fig. 2B). In contrast, in apical supernatants from DMM-BMECs active virion concentration increased from 1 to 72 hpi indicating that DMM-BMECs were productively infected by SARS-CoV-2 (Fig. 2B). With higher inoculum the released virion levels increased, but never reached the levels of the original inoculates. The released amounts of infectious virions by DMM-BEMCs also remained 10-100-fold lower compared to those obtained from Calu-3 cells, although they were infected with an up to 250-fold higher inoculum suggesting lower infectibility of DMM-BMECs compared to Calu-3 cells. Whether DMM-BMECs also released virions to the basolateral side could not be reliably determined as the coated filter membrane with 0.4 μm pore-size did not allow for unhindered SARS-CoV-2 diffusion (data not shown). SARS-CoV-2 infection did not induce any overt cytopathogenic effects in DMM-BMECs, since the monolayers and junctional localization of E-cadherin remained intact (Fig. 2A). The frequency of infection of DMM-BMECs at 72 hpi increased with higher inoculate concentration, with differences depending on the respective hiPSC donor (Fig. 2C-D). Although ACE2 could not be reliably detected in DMM-BMECs, co-incubation of anti-ACE2 blocking antibody with SARS-CoV-2 resulted in a significantly lower SARS-CoV-2 infection as shown by the decreased frequency of SARS-CoV-NP^+^ DMM-BMECs (Fig. 2E). This suggests that SARS-CoV-2 infection of DMM-BMECs occurred mainly in an ACE2-dependent manner and that very low expression levels, i.e. below the detection levels of Western Blotting and IF staining, were sufficient. In contrast, in EECM-BMECs and BPLCs the absence of ACE2 and TMPRSS2 expression might account for their inability to be infected with SARS-CoV-2. Overall, our results suggest that the BBB is an unlikely target of SARS-CoV-2 infection.

### Lack of interaction of SARS-CoV-2 with EECM-BMECs under inflammatory conditions

**Fig. 3:**
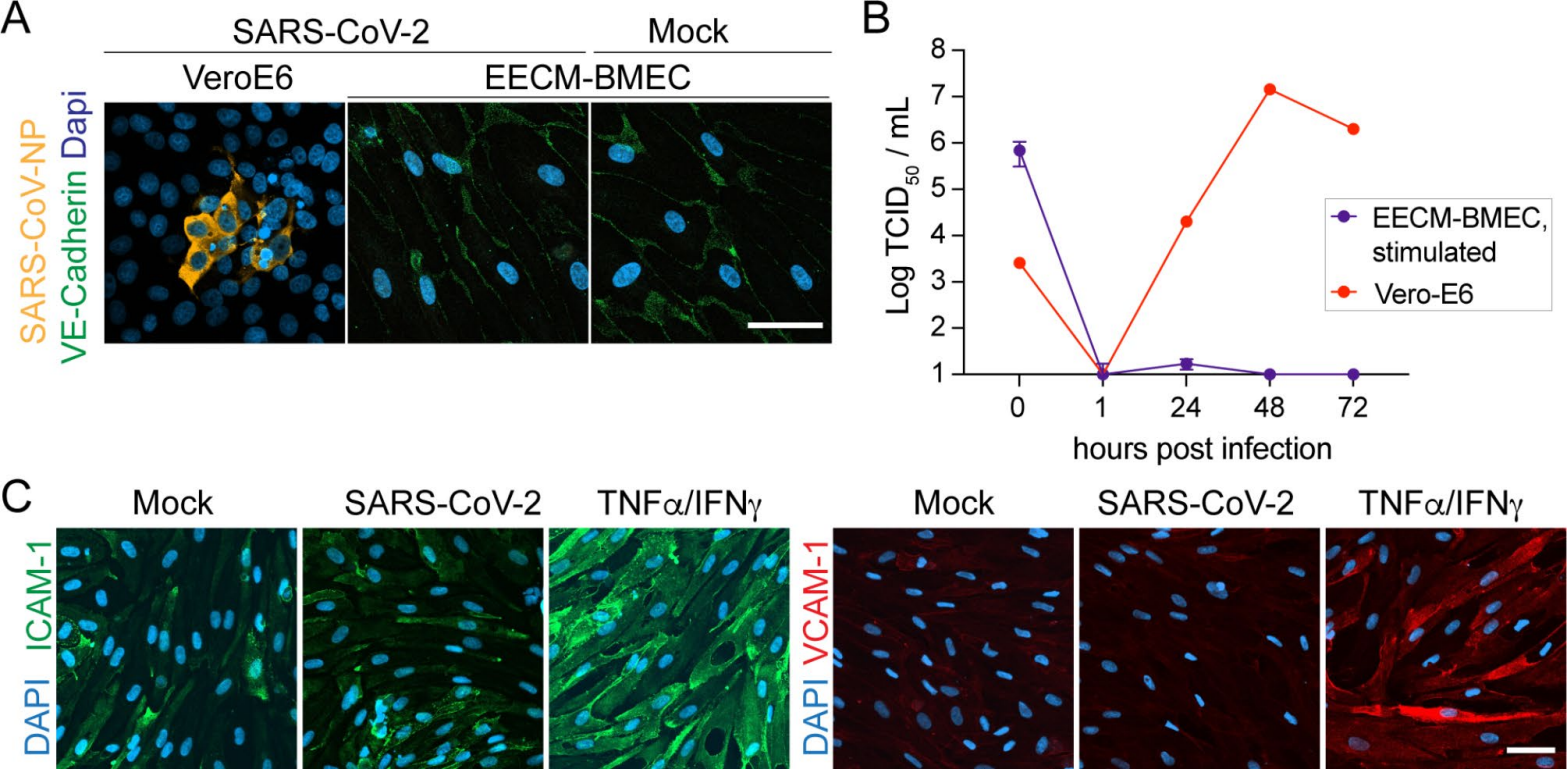
Inflammatory conditions do not alter interaction of SARS-CoV-2 with EECM-BMECs. **(A)** Representative confocal images from immunofluorescence staining for SARS-CoV-2 NP (orange) and VE-cadherin (green) 72 hpi with SARS-CoV-2 at 50’000 TCID_50_/ well or Mock in EECM-BMECs, which were pre-stimulated with TNFα (1 ng/mL) and IFNγ (20 IU/mL) for 20 h, are shown. Nuclei were stained with DAPI (blue). Three iPSC-clone-derived EECM-BMECs with 2 replicates per condition were tested. As a positive control VeroE6 cells 72 hpi with SARS-CoV-2 at 400 TCID_50_/ well is shown. Scale bar = 50 μm. **(B)** Quantification of released virions into the supernatant 1–72 hpi with SARS-CoV-2 by TCID_50_ assay. Measurement was done in duplicates from a total of 3 experiments with EECM-BMECs (each time a different iPSC clone) and 1 experiment with VeroE6 cells. **(C)** Representative confocal images of immunofluorescence staining for cell adhesion molecules VCAM-1 (red) and ICAM-1 (green) in EECM-BMECs after 24 h inoculation with Mock, SARS-CoV-2 or as a positive control stimulation with TNFα (1 ng/mL) and IFNγ (20 IU/mL) are shown. Nuclei were stained with DAPI (blue). Scale bar = 50 μm. 3 iPSC clone-derived EECM-BMECs with 2 replicates per condition were tested

Markers of endothelial activation in the circulation were reported to be increased in COVID-19 patients and to correlate with disease severity such as soluble cell adhesion molecules including VCAM-1 [29], P-selectin [31, 76] and E-selectin [34], as well as vWF and angiopoetin-2 [31, 77] and this could be due to systemic inflammation. To address whether inflammation-mediated activation of BBB endothelial cells is a prerequisite for infection by SARS-CoV-2, we stimulated EECM-BMEC with the pro-inflammatory cytokines TNFα/IFNγ prior to infection. Cytokine stimulation of EECM-BMECs failed to increase susceptibility of EECM-BMECs to SARS-CoV-2 infection, since 72 hpi no SARS-CoV-NP^+^ cells were detected (Fig. 3A) and no active virions were present in the supernatant as determined by TCID_50_ assay (Fig. 3B). Lack of infection could still be due to absence of endothelial *ACE2* expression also in the stimulated condition (Fig. S2E, Additional file 1). Previously, ACE2 was proposed to be a type-I interferon (IFN) stimulated gene in airway epithelial cells [78] and upregulation of ACE2 at mRNA and protein level in pulmonary microvascular endothelial cells after treatment with type-I IFNs was reported [79]. We did not find altered ACE2 protein expression in EECM-BMECs 24 h after treatment with IFN-α (Fig. S2F, Additional file 1).

Vice versa, exposure to SARS-CoV-2 or parts of it without active infection could activate innate immune pathways in endothelial cells leading to their activation [80–83] which may initiate subsequent pathomechanisms. We therefore determined cell surface staining of the adhesion molecules VCAM-1 and ICAM-1 in EECM-BMECs after inoculation with SARS-CoV-2 for 24 h. Stimulation with TNFα and IFNγ resulted in significant upregulation of these adhesion molecules as assessed by IF staining, but in SARS-CoV-2 exposed EECM-BMECs no upregulation of VCAM-1 and ICAM-1 was observed as compared to mock-treated samples (Fig. 3C) suggesting that in the absence of additional soluble or cellular blood components or infected tissue-resident cells SARS-CoV-2 does not activate BBB endothelial cells. Overall, a proinflammatory environment is not a prerequisite for EECM-BMECs to be infected by SARS-CoV-2, and neither can SARS-CoV-2 directly induce an inflammatory phenotype of EECM-BMECs.

### HIBCPP cells were productively infected by SARS-CoV-2

**Fig. 4:**
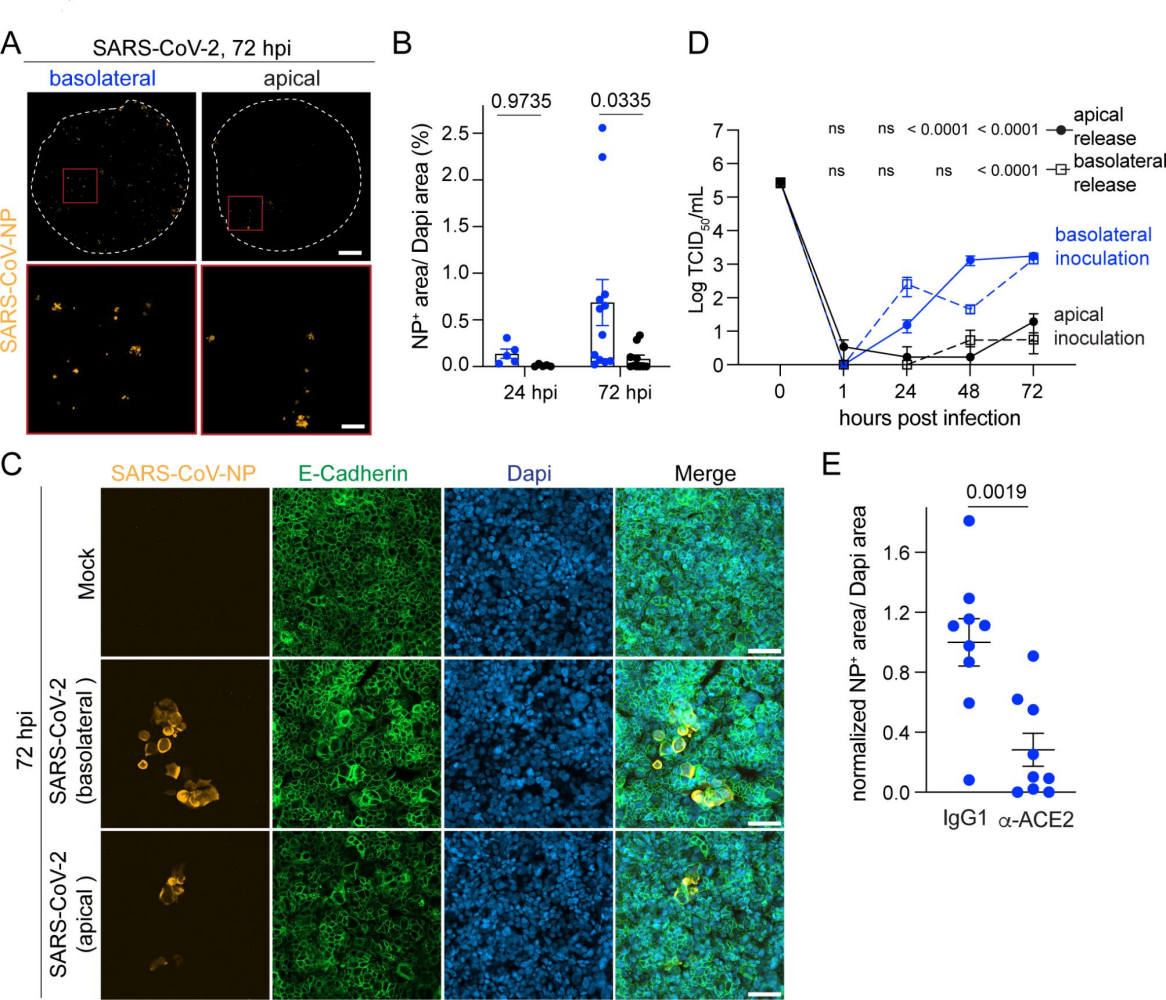
SARS-CoV-2 infects HIBCPP preferentially from the basolateral side. **(A)** Representative tile stack images from immunofluorescence staining for SARS-CoV-nucleocapsid protein (NP) (orange) of whole filter inserts with HIBCPP cells 72 hpi with SARS-CoV-2 at 100’000 TCID_50_/filter insert from basolateral and apical side are shown. White dashed circle indicates border of the filter inserts, red box delineates inset. Scale bar = 1 mm and for inset 200 μm. **(B)** Quantification of NP^+^ area normalized to DAPI^+^ area 24 and 72 hpi with SARS-CoV-2. Each dot represents a replicate from in total four experiments. P-values were determined by two-way Anova followed by Sidàk test to correct for multiple comparison. **(C)** Representative confocal images from immunofluorescence staining of SARS-CoV-NP (orange) and E-Cadherin (green) 72 hpi with SARS-CoV-2 from basolateral and apical side or Mock are shown. Nuclei were stained with DAPI (blue). Scale bar = 50 μm. **(D)** Quantification of released virions into the basolateral and apical supernatant 1–72 hpi with SARS-CoV-2 by TCID_50_ assay. Measurement was done in duplicates or triplicates from a total of 4 experiments. P-values were determined using 2-way Anova followed by Tukey’s test to correct for multiple comparison and are indicated for the comparison of the supernatant from the respective compartment from basolateral versus apical infection. **(E)** Quantification of NP^+^ area normalized to DAPI^+^ area 72 hpi with SARS-CoV-2 at 100’000 TCID_50_/filter insert from basolateral side in presence of anti-ACE2 blocking or IgG control antibody. Data is normalized to IgG control. Each dot represents a replicate from in total 3 experiments. P-value was calculated using unpaired T-test with Welch’s correction

Barrier dysfunction in COVID-19 patients could also occur at the level of the BCSFB. To assess whether ChP epithelial cells are susceptible to SARS-CoV-2 infection, we inoculated HIBCPP cells with SARS-CoV-2 either from the basolateral or apical side corresponding to the blood or CNS facing compartment, respectively. At 24 hpi and increasingly at 72 hpi, SARS-CoV-NP^+^ HIBCPP cells were detected and at significantly higher frequency after basolateral compared to apical SARS-CoV-2 inoculation (Fig. 4A-C). Similarly, a gradual increase of active virions was found in the basolateral and apical supernatants up to 72 hpi after basolateral SARS-CoV-2 infection, while after apical inoculation virus titers in both compartments only increased marginally (Fig. 4D). SARS-CoV-2 concentrations released by HIBCPP cells stayed < 100-fold below the initial inoculum, which could be due to a low initial infection rate, low virus replication and/or a low subsequent re-infection efficacy. Still, these results show that HIBCPP cells were productively infected by SARS-CoV-2 with a preference from the basolateral side, which is the blood facing side of the BCSFB cells. This polarity of infection could not be explained by polarized expression of ACE2 or TMPRSS2 at the basolateral side as both molecules were rather found at the apical side of HIBCPP cells (Fig. 1D, Fig. S2D, Additional file 1). Productive infection of HIBCPP cells from the basolateral side did however involve ACE2 at least in part, since basolateral SARS-CoV-2 infection of HIBCPP cells in the presence of anti-ACE2 blocking antibody significantly reduced the infection frequency at 72hpi despite high inter-replicate variability (Fig. 4E). In conclusion, these results demonstrate that ChP epithelial cells could indeed be a target of SARS-CoV-2 and this preferentially in the case SARS-CoV-2 reaches the BSFB from the blood side.

### ACE2 is selectively localized to the ChP stroma

**Fig. 5:**
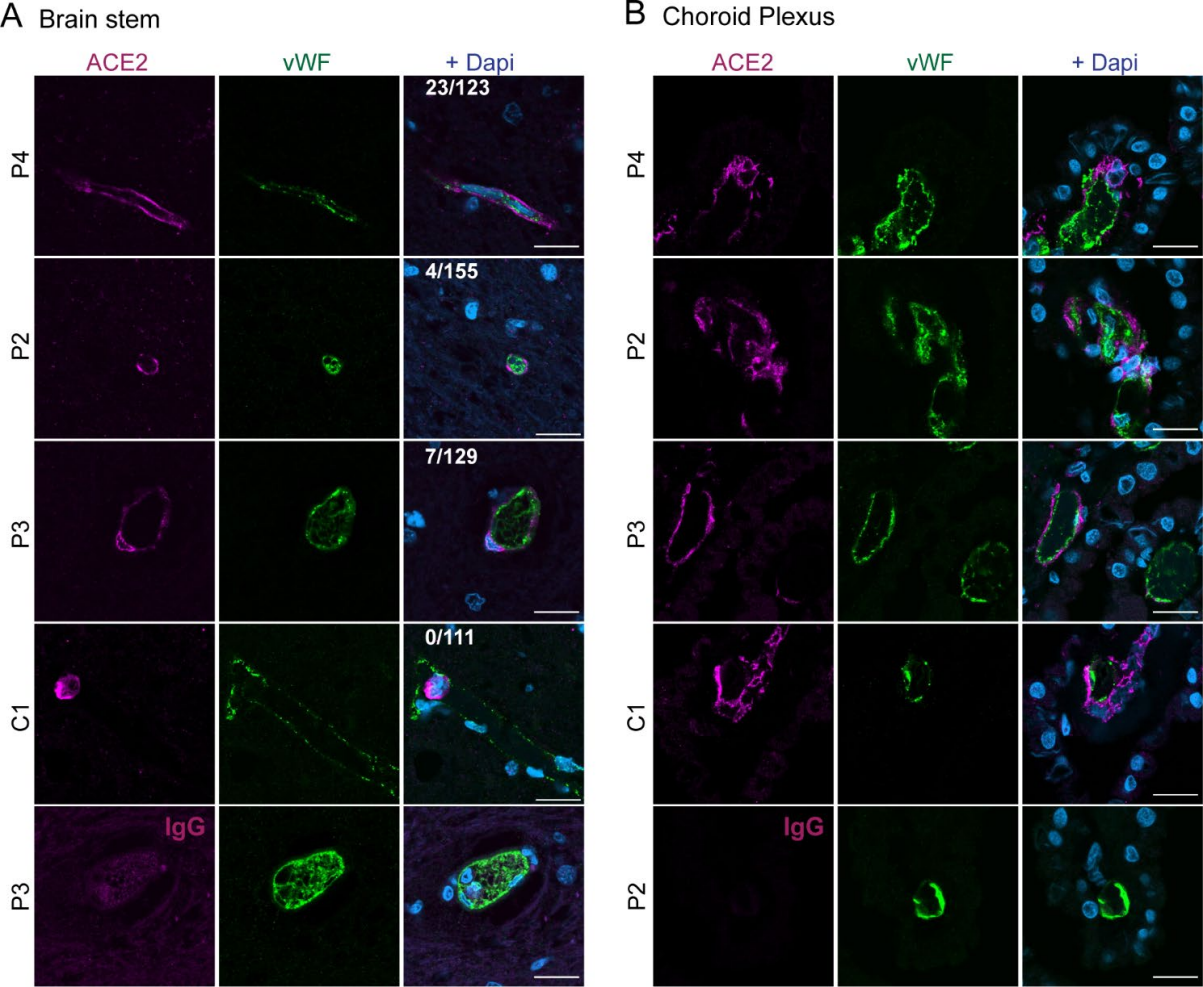
ACE2^+^ cells localized next to endothelial cells in brain stem and ChP in COVID-19 patients. **A-B)** Representative confocal images from immunofluorescence staining of ACE2 (magenta) and von Willebrand factor (vWF, green) of brain stem (medulla oblongata) **(A)** and choroid plexus **(B)** from 3 COVID-19 patients and 1 control. Nuclei were stained with DAPI (blue). Scale bar = 20 μm. The numbers indicated in the merged image of **A** indicate the number of vWF^+^ vessels with ACE2 signal / total number of vWF^+^ vessels. Quantification was done from 15 images per patient

Due to controversial results concerning the presence of ACE2 expression in parenchymal endothelial cells of human brain tissues [26, 71, 84], we next assessed detection of ACE2 by IF staining in brain stem (medulla oblongata) and ChP sections from COVID-19 patients. In brain stem, ACE2 immunoreactivity was found adjacent to but not co-localized with 8.9 ± 5.0% of vWF^+^ endothelial cells of smaller and larger blood vessels (Fig. 5A). These results suggest that in the brain stem, a subset of pericytes or perivascular macrophages rather than brain endothelial cells express ACE2. In one non-COVID-19 control patient, no vascular ACE2 immunoreactivity was found, but blood cells fixed within the blood vessel lumen showed strong ACE2 immunostaining (Fig. 5A, C1). Also, in the COVID-19 tissues ACE2^+^ blood cells were frequently found within blood vessels of the brain stem (data not shown). In the ChP, strong ACE2 immunostaining was observed within the ChP stroma, often in close proximity to vWF^+^ endothelial cells and surprisingly, ChP epithelial cells did not stain positive for ACE2 (Fig. 5B). These results indicate that cells in the ChP stroma such as macrophages, pericytes and/or fibroblasts are the predominant cells showing ACE2 immunoreactivity.

### ChP epithelial cells of COVID-19 patients harbor ***SARS-CoV-2 S*** transcripts

**Fig. 6:**
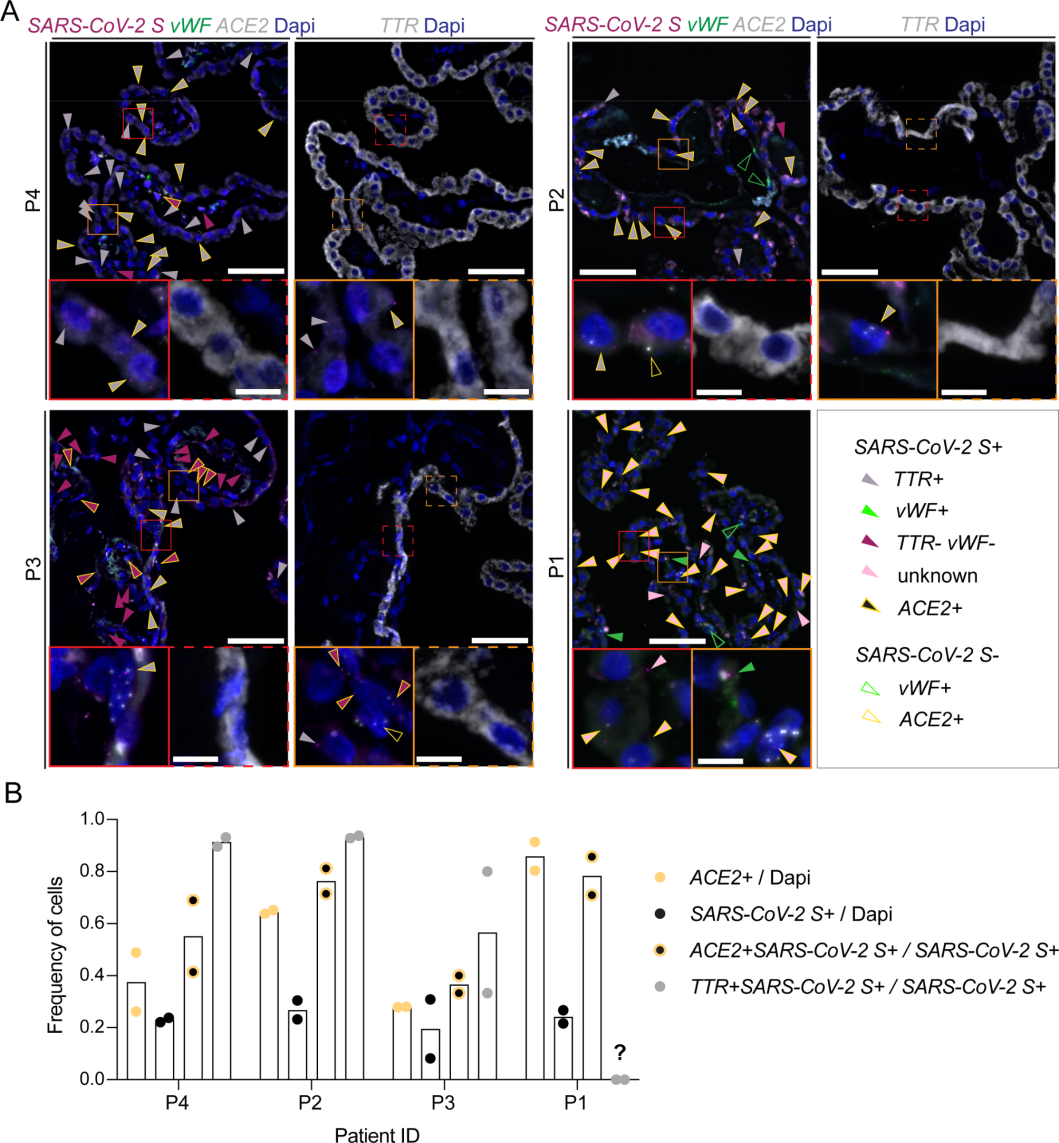
SARS-CoV-2 RNA is detected in ChP epithelial cells in COVID-19 patients. **(A)** Representative images from fluorescent RNA in situ hybridization for *SARS-CoV-2 S* (magenta), *vWF* (green) and *ACE2* (grey) or *transthyretin* (*TTR, grey*) of ChP from 4 different patients are shown. Nuclei were stained with DAPI (blue). All images are maximum intensity Z projections. Red and orange boxes (full and dashed line) define area of inset. Scale bars = 50 μm and 20 μm for inset. Arrow heads mark all identified cells positive for transcripts as indicated in the legend. *ACE2*^*+*^*SARS-CoV-2 S*^*−*^ cells (yellow-lined, transparent arrowhead) were only marked in the inset pictures. **(B)** Quantification of number of *ACE2*^*+*^ and *SARS-CoV-2 S*^*+*^ cells normalized to number of DAPI^+^ nuclei per section and of *ACE2*^*+*^*SARS-CoV-2 S*^*+*^ cells and of *SARS-CoV-2*^*+*^*TTR*^*+*^ epithelial cells normalized to the total number of *SARS-CoV-2 S* transcript positive cells. 2 sections per patient were analyzed, for patient P1 the corresponding area in the consecutive section stained for *TTR* could not be identified

To corroborate our in vitro observations that the BCSFB rather than the BBB is a cellular target of SARS-CoV-2, we employed multiplex RNA in situ hybridization for *SARS-CoV-2 spike (S)* transcripts in ChP obtained from COVID-19 patients, who died due to respiratory failure 2–3 weeks post symptom onset. In addition, RNA probes for *ACE2* as the main entry factor and *vWF* as a marker for endothelial cells and on consecutive sections *transthyretin* (*TTR*) as a marker for ChP epithelial cells were applied (Fig. 6A). In all 4 assessed COVID-19 tissues, *SARS-CoV-2 S* transcripts were observed in 20–30% of cells per image, from which the majority corresponded to *TTR*^*+*^ ChP epithelial cells, except in one patient (P3) in which an equal number of not further defined stromal cells were *SARS-CoV-2 S*^*+*^ (Fig. 6A-B and Fig. S3, Additional file 1). Occasionally, *vWF*^+^ endothelial cells were positive for *SARS-CoV-2 S* (Fig. 6A). The frequency of *SARS-CoV-2*^*+*^ cells, which were also characterized by *ACE2* co-expression, reflected the frequency of *ACE2*^*+*^ cells within the total cell population. There was no correlation between total numbers of *ACE2* and the total number of *SARS-CoV-2*^*+*^ cells per section (Fig. 6B). In contrast to the results obtained with immunostaining, in the majority of *TTR*^+^ ChP epithelial cells *ACE2* transcripts were observed. Only few *ACE2* puncta were detected per ChP epithelial cells suggesting low baseline gene expression, which thus might also be below the detection limit of immunostaining protocols for ACE2. Although it is not known whether the analyzed patients suffered from CNS-related symptoms, these data underscore that ChP epithelial cells can be a target of SARS-CoV-2 in severely ill COVID-19 patients.

## Discussion

The frequent occurrence of a broad range of CNS-related symptoms observed in patients during and after COVID-19 [1, 2, 4] raised the question of whether CNS-resident or CNS-associated cells are a direct target of SARS-CoV-2 infection [85]. Here, we investigated the susceptibility of cells constituting the BBB and the BCSFB of the ChP to SARS-CoV-2 infection and found that hiPSC-derived brain microvascular endothelial cells (EECM-BMECs) and brain pericytes (BPLCs) were refractory to infection. In contrast, hiPSC-derived BMECs (DMM-BMECs) showing a mixed endothelial/ epithelial phenotype and ChP epithelial cells forming the BCSFB (HIBCPP) were permissive to productive SARS-CoV-2 infection. Analysis of ChP sections from COVID-19 patients, who succumbed to respiratory failure, confirmed infection of ChP also in vivo. Our study thus highlights that the ChP and its BCSFB rather than the BBB are susceptible to direct SARS-CoV-2 infection. Future studies should therefore consider a potential role of the ChP as a major route of infection leading to neurological sequelae associated with COVID-19.

The resistance of EECM-BMECs to SARS-CoV-2 infection may be due to the lack of detectable expression of ACE2, the main SARS-CoV-2 entry receptor [60, 61]. EECM-BMECs showed high expression of the proposed additional entry receptors BSG [64] and NRP1 [62] suggesting that these alone are not sufficient to mediate SARS-CoV-2 entry into EECM-BMECs. The lack of ACE2 in and no SARS-CoV-2 infection of EECM-BMECs is in accordance with several other in vitro studies, which recently reported absence of productive infection of primary endothelial cells isolated from various vascular beds including the lung and the brain, iPSC-derived endothelial cells and the immortalized BMEC line hCMEC/D3, except when *ACE2* over-expression was introduced by lentiviral transduction [72, 80, 83, 86, 87].

Presence of ACE2 in human brain endothelial cells is controversial as immunohistochemistry on human post-mortem brain samples showed an occasional vascular staining pattern of ACE2, but it was not clear whether the signal stems from endothelial or mural cells [88, 89]. Some studies reported a more frequent vascular ACE2 immunoreactivity in various brain regions of COVID-19 patients than in control patients [71, 90], others found it to be more frequent in the frontal cortex of patients with hypertension or dementia compared to controls [84] suggesting that brain parenchymal vascular ACE2 expression may depend on the underlying pathological condition. Here, we observed ACE2 immunoreactivity in the vicinity of vWF^+^ endothelial cells in a small subset of vessels in the brainstem of COVID-19 patients indicating that ACE2 proteins are localized to vascular mural cells such as pericytes but not endothelial cells. Indeed, a previous report showed ACE2 colocalization with PDGFRb^+^ pericytes rather than CD31^+^ endothelial cells in human prefrontal cortex [26]. This is in line with recent single nucleus RNA sequencing data obtained from microvessels isolated from fresh-frozen post-mortem hippocampus and frontal cortex from individuals without cognitive impairment and Alzheimer’s disease patients, in which *ACE2* transcripts were not detected in arterial, capillary and venous brain endothelial cells, but in a small percentage of pericytes and vascular smooth muscle cells [74]. Similarly a recent report analysing several single cell RNA-sequencing data sets obtained from adult mouse brains found *ACE2* expression in a large fraction of pericytes and to a lesser extent in venous smooth muscle cells but not in arterial, capillary and venous endothelial cells, which was also confirmed by immunoreactivity [73]. Together these data show that absence of ACE2 expression in brain endothelial cells in vitro is not a cell culture artifact and indicates that vascular mural cells such as pericytes are a more likely target of SARS-CoV-2 infection.

In our hands, BPLCs derived from several hiPSC lines did not show ACE2 expression and were not infected by SARS-CoV-2. This stands in apparent contrast to previous observations, which showed expression of *ACE2* in BPLCs differentiated using the same protocol as employed by us in addition to productive infection leading to up to 60% of infected BPLCs at 72 hpi as visualized by immunoreactivity for spike protein [75]. As BPLCs were differentiated from different hiPCS in the previous and present study, it is tempting to speculate that *ACE2* expression in brain pericytes may be variable in different individuals. Interestingly, primary heart pericytes derived from patients with congenital heart defects displayed differential levels of ACE2 protein by Western Blotting and only 2 out of 6 pericyte isolations were susceptible to SARS-CoV-2 infection in vitro highlighting the potential of donor-to-donor variability in susceptibility of pericytes to SARS-CoV-2 infection [91]. Our results question brain pericytes to be a major cellular target of SARS-CoV-2. However, a previous study suggested that in patients with high blood pressure minor vascular lesions at the level of the endothelial layer may allow blood-borne SARS-CoV-2 to infect pericytes [73]. Another recent study observed accumulation of *SARS-CoV-2* RNA by in situ hybridisation in the perivascular spaces in the olfactory bulb and the frontal lobe in a subset of their patient cohort [92]. Although this study did not detect infection of vascular cells, it points to the possibility of SARS-CoV-2 reaching perivascular spaces and thus the option for infecting vascular mural cells from the brain side. This may allow for pericyte-associated neuropathological mechanism as recently proposed based on findings of reduced capillary diameters in ex vivo Golden Syrian hamster and human brain slice cultures in response to treatment with receptor binding domain of spike protein or spike protein pseudo-typed virus in combination with angiotensin 2 (substrate of ACE2). This study suggested that SARS-CoV-2 spike protein-mediated internalization of ACE2 in pericytes could lead to locally reduced cerebral capillary blood flow or constriction of individual capillaries, which may cause cognitive deficits [93].

Mimicking systemic inflammation as it occurs during COVID-19 by pre-stimulation of EECM-BMECs with TNFα/IFNγ still failed to allow for productive infection of EECM-BMECs with SARS-CoV-2. This is likely attributed to the lack of ACE2 expression, which was not upregulated in EECM-BMECs under inflammatory conditions, while high expression of additional proposed entry receptors NRP1 and BSG was not sufficient to mediate SARS-CoV-2 infection.

Shed spike protein in the circulation of COVID-19 patients [94] or SARS-CoV-2 without cell entry could trigger innate immune pathway activation in the endothelium. In vitro studies showed that incubation with spike protein 1 led to activation of primary fetal BMECs [84] and a dermal endothelial cell line presumably through ACE2 [82], and human umbilical vein endothelial cells (HUVECs) through α5β1 integrin [81]. Although these studies administered recombinant spike protein 1 only in the low nanomolar range, this corresponds to at least a 100’000-fold higher concentration of spike protein added to the cells when compared to addition of SARS-CoV-2 virions. This difference in spike protein concentration might be a reason for the discrepancy between response to spike protein 1 alone and absence of induction of expression of cell adhesion molecules ICAM-1 and VCAM-1 in EECM-BMECS upon prolonged exposure to SARS-CoV-2. On the contrary, SARS-CoV-2 inoculation was shown to be sufficient to trigger activation of iPSC-derived peripheral-like endothelial cells through activation of the plasma membrane bound pattern recognition receptor TLR4 [80] and of commercially available primary lung microvascular endothelial cells through unknown mechanisms [83]. Our results speak against that SARS-CoV-2 alone can induce an inflammatory phenotype of BBB endothelial cells.

Interestingly, in contrast to EECM-BMECs, DMM-BMECs were productively infected by SARS-CoV-2 in an ACE2-dependent manner although *ACE2* mRNA expression levels were very low and ACE2 was inconsistently detected at protein level. This indicates that even very low *ACE2* expression levels may be sufficient to mediate SARS-CoV-2 cell entry. This agrees with an earlier study, which reported productive ACE2-dependent SARS-CoV-2 infection in iPSC-derived BMECs that were differentiated using the unconditioned medium method (UMM) [95]. UMM-BMECs are phenotypically more similar to DMM-BMECs than EECM-BMECs [42, 96, 97]. DMM- and UMM-BMECs exhibit excellent barrier properties and have been well characterized with respect to their expression of BBB specific transporters and carriers [44, 97, 98]. It has also been shown that they display a mixed endothelial/ epithelial transcriptome profile [96]. We have previously shown that DMM- and UMM-BMECs lack a mature immune phenotype with expression of all endothelial adhesion molecules involved in immune cell interactions [42]. In context of COVID-19 research we therefore consider EECM-BMECs as the more appropriate cellular model for BBB endothelial cells as they are more suitable to study the interaction with cellular and humoral immune components. Overall, distinguished susceptibility of EECM- versus DMM- and UMM-BMECs to SARS-CoV-2 infection highlights the need for careful consideration of the choice of in vitro BBB model to study vascular pathogenesis in COVID-19.

Besides the BBB, SARS-CoV-2 could also reach the BCSFB from either the vascular side or via the ventricular CSF. Our study suggests that ChP epithelial cells forming the BCSFB are a cellular target of SARS-CoV-2. This is in agreement with previous in vitro reports, which found productive infection with SARS-CoV-2 of hPSC-derived ChP organoids [15, 99], and hiPSC-derived ChP epithelial cell aggregates [16], as well as by the fact that ChP epithelia express ACE2 and TMPRSS2 in situ [68–71]. Notably, HIBCPP cells showed preferential infection from the basolateral rather than the apical side. This cannot be explained by expression of ACE2 and TMPRSS2, which were rather found polarized to the apical side of HIBCPP cells. Also in ChP organoids ACE2 was found to primarily localize at the apical surface of ChP epithelial cells [15], as well as in ChP epithelial cells in mouse brain [70]. Whereas RNA in situ hybridisation clearly demonstrated the presence of *ACE2* transcripts, we did not detect presence of ACE2 protein in ChP epithelial cells by immunoreactivity and instead found that ChP stromal cells exhibited a strong ACE2 immunoreactivity. Interestingly, Piras et al. reported a similar staining pattern of ACE2 in fetal and adult ChP by IHC using a different anti-ACE2 antibody [100]. In line with our immunoreactivity data, which showed absence of ACE2 in ChP endothelial cells, in both primary and immortalized human ChP endothelial cells, ACE2 was neither detected at the RNA nor at the protein level (personal communication with HS).

Preferential infection of ChP epithelial cells from the basolateral side suggests that the BCSFB is more vulnerable to SARS-CoV-2 infection from the periphery via the blood than from within the CNS via the CSF. Efflux routes of solutes and possibly cells from the subarachnoid space through the cribriform plate into the nasal mucosa along the perineurium of the olfactory nerves are assumed to occur due to discontinuous arachnoid mater at the level of the olfactory bulb, where the olfactory nerves leave [101]. Thus, a possibility of SARS-CoV-2 to reach the CNS could be leakage from the highly infected nasal epithelium into the perineural space along olfactory nerves and further into the subarachnoid space around the olfactory bulb and subsequently into the CSF. On the other hand, lymphatic vessels enwrapping the olfactory nerve bundles and connecting the olfactory bulbs with the nasal submucosa through the cribriform plate may allow for constant CSF out-flow, which could counteract leakage of viral particles from the nasal cavity into the CNS [102]. In a recent report, *SARS-CoV-2* RNA was detected by RNA in situ hybridisation in the interstitial spaces close to the olfactory nerves, but not beyond a certain perineural fibroblast population, which may suggest that SARS-CoV-2 does not enter the CSF via the olfactory nerve perineurium [92].

Two studies did not detect SARS-CoV-2 transcripts in ChP from severely ill COVID-19 patients by SARS-CoV-2 targeted RNA-sequencing of 5 [103] and bulk RNAseq of 7 patients [69], respectively, although single nucleus RNA-sequencing demonstrated that the ChP epithelial cells from COVID-19 patients acquired a molecular signature of an inflamed state compared to healthy controls [69]. So far, a case study by Fuchs et al. reported the presence of spike protein transcript by RNA in situ hybridisation in ChP epithelial and ventricular ependymal cells in two COVID-19 patients [57] and another report found SARS-CoV-2 spike protein in ChP epithelial and a few ependymal cells lining the lateral ventricle by immunoreactivity in a COVID-19 deceased paediatric patient [104]. Analyzing ChP tissue from 4 COVID-19 patients, which succumbed to respiratory failure, by multiplex RNA in situ hybridisation, we found *SARS-CoV-2 S* transcripts predominantly in *TTR*^+^ epithelial cells, to a lesser extent in not further defined ChP stromal cells and very rarely in *vWF*^+^ endothelial cells. Of note, one of the patients was previously reported [57]. Independent of the cell type, signals for the *SARS-CoV-2* spike protein transcript were very low with positive cells showing between 1 and 3 puncta corresponding to 1–3 target RNAs. This might be due to the post-symptom onset interval of 2–3 weeks, a time point, in which usually active SARS-CoV-2 virions are cleared even from the respiratory tract [105]. This data does not allow to decide if the low signal per cell derives from left over virus particles in cells, which supported SARS-CoV-2 replication before, or just shows the possibility of occasional infection of cell types within the brain without replication. Considering the strong ACE2 immunoreactivity in the ChP stroma, it could be that initial infection of stromal cells leads to locally high virus titers in the ChP, which may result in infection of ChP epithelial cells. To what extent ChP epithelial cells support SARS-CoV-2 replication awaits further investigation. The rarely detected low levels of SARS-CoV-2 RNA in CSF from alive as well as deceased COVID-19 patients rather speaks against significant SARS-CoV-2 replication in ChP epithelial cells followed by release into the CSF [12, 13, 92, 106–108]. Furthermore, future studies need to assess potential involvement and vulnerability of the ChP during COVID-19-associated neuropathology such as to what extent infection of ChP stromal and epithelial cells infers with the epithelial cells function as a barrier, a CSF production site and/or release of pro-inflammatory cytokines and chemokines into the CSF leading to neuroinflammation.

## Conclusions

This study suggests that ChP epithelial cells, the constituent of the BCSFB rather than BBB endothelial cells could be a target of SARS-CoV-2 infection. Alteration in BCSFB of the ChP could thus at least in part contribute to CNS-related symptoms during and after COVID-19.

### Electronic supplementary material

Below is the link to the electronic supplementary material.

Supplementary Material 1

## Data Availability

All data generated or analysed during this study are included in this published article and its supplementary information file (Additional file 1).

## Abbreviations

SARS-CoV-2: Severe acute respiratory syndrome corona virus 2
COVID-19: Corona virus disease 2019
CNS: Central nervous system
hiPSC: Human induced pluripotent stem cells
BMEC: Brain microvascular endothelial cells
BBB: Blood-brain barrier
ChP: Choroid plexus
CSF: Cerebrospinal fluid
BCSFB: Blood-cerebrospinal fluid barrier
EECM: Extended endothelial culture method
EPC: Endothelial progenitor cell
SMLC: Smooth muscle-like cell
DMM: Defined medium method
BPLCs: Brain pericyte-like cells
TCID_50_: Median tissue culture infectious dose
BSL3: Biosafety level 3
hpi: Hours post infection
IF: Immunofluorescence
TEER: Transendothelial/-epithelial electrical resistance
NP: Nucleocapsid protein
MFI: Mean fluorescent intensity
S: Spike
UMM: Unconditioned medium method
