# Supplementary material for: SARS-CoV-2 infects epithelial cells of the blood-cerebrospinal fluid barrier rather than endothelial cells or pericytes of the blood-brain barrier
Source: Fluids Barriers CNS. 2023 Oct 24;20:76. doi: 10.1186/s12987-023-00479-4 (PMC10598911; doi:10.1186/s12987-023-00479-4)
Supplement: Supplementary file 1 — Supplementary Material 1 [file 12987_2023_479_MOESM1_ESM.docx]

**Additional file 1**

**SARS-CoV-2 infects epithelial cells of the blood-cerebrospinal fluid barrier rather than endothelial cells or pericytes of the blood-brain barrier**

Chiara Stüdle^1^, Hideaki Nishihara^1,2^, Sven Wischnewski^3^, Laila Kulsvehagen^4^, Sylvain Perriot^5^, Hiroshi Ishikawa^6^, Horst Schroten^7^, Stephan Frank^8^, Nikolaus Deigendesch^8^, Renaud Du Pasquier^5,9^, Lucas Schirmer^3,10,11^, Anne-Katrin Pröbstel^4^, Britta Engelhardt^1^

^1^Theodor Kocher Institute, University of Bern, Bern, Switzerland;

^2^Current address: Yamaguchi University, Department of Neurotherapeutics, Yamaguchi, Japan;

^3^Department of Neurology, Medical Faculty Mannheim, Heidelberg University, Mannheim, Germany;

^4^Departments of Neurology, Biomedicine and Clinical Research, and Research Center for Clinical Neuroimmunology and Neuroscience Basel (RC2NB), University Hospital Basel and University of Basel, Basel, Switzerland;

^5^Laboratory of Neuroimmunology, Neuroscience Research Centre, Department of Clinical Neurosciences, Lausanne University Hospital (CHUV) and University of Lausanne, Lausanne, Switzerland;

^6^Laboratory of Clinical Regenerative Medicine, Department of Neurosurgery, University of Tsukuba, Tsukuba, Ibaraki, 305-8575, Japan;

^7^Pediatric Infectious Diseases, Department of Pediatrics, Medical Faculty Mannheim, Heidelberg University, Mannheim, Germany;

^8^Pathology, Institute of Medical Genetics and Pathology, University Hospital Basel and University of Basel, Switzerland;

^9^Service of Neurology, Department of Clinical Neurosciences, Lausanne University Hospital (CHUV) and University of Lausanne, Lausanne, Switzerland;

^10^Center for Translational Neuroscience and Institute for Innate Immunoscience, Medical Faculty Mannheim, Heidelberg University, Mannheim, Germany;

^11^Interdisciplinary Center for Neurosciences, Heidelberg University, Heidelberg, Germany

**Key words:** SARS-CoV-2, blood-brain barrier, blood-cerebrospinal fluid barrier, hiPSC-derived brain microvascular endothelial cells, choroid plexus epithelial cells

**Corresponding authors:**

Prof. Dr. Britta Engelhardt and Dr. Chiara Stüdle

Theodor Kocher Institute

University of Bern

Freiestr. 1

CH 3012 Bern

Switzerland

E-Mail: britta.engelhardt@unibe.ch and chiara.stuedle@unibe.ch


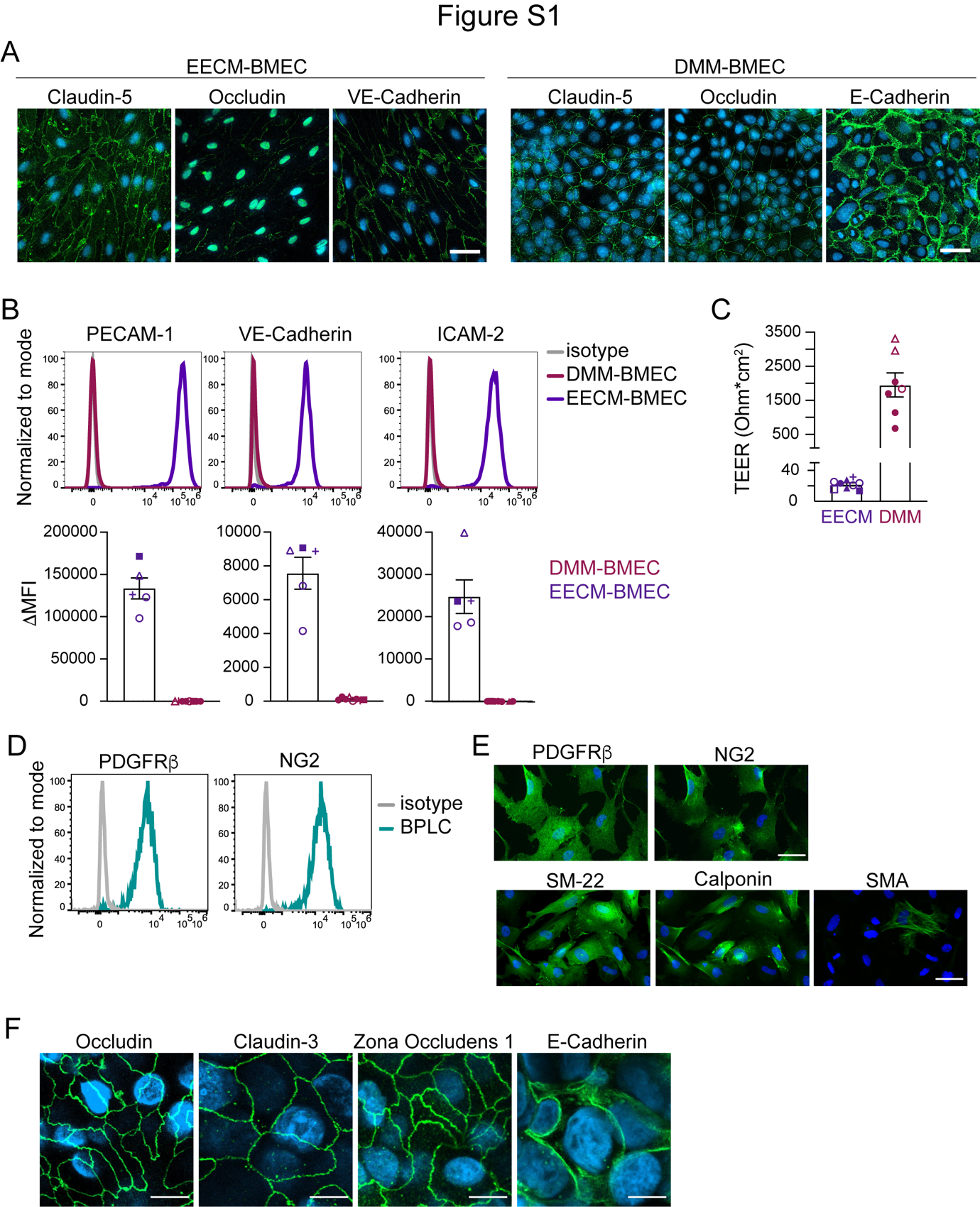


**Fig. S1. Characterization of human *in vitro* brain barrier models. A)** Representative confocal images from immunofluorescence staining for Claudin-5 (green), Occludin (green) and VE-Cadherin (green) in EECM-BMECs at day 6 after seeding on filter inserts or E-Cadherin (green) in DMM-BMECs at day 10 of differentiation. Nuclei were stained with DAPI (blue). Scale bar = 50 μm. **B)** Top: Representative histogram overlays of flow cytometry analysis for PECAM1, VE-Cadherin and ICAM-2 on EECM-BMECs (purple) and DMM-BMECs (dark red) are shown. Isotype control is in grey. Below: Quantification of flow cytometry data as geometric mean fluorescence intensity (MFI) from which the values of isotype controls were subtracted is shown. Each symbol represents an independent differentiation and each symbol type corresponds to an iPSC clone (EECM-BMECs: 5 differentiation with 4 clones, DMM-BMECs: 8 differentiations with 4 clones). Data is shown as average with standard error mean. **C)** Transendothelial/epithelial electrical resistance (TEER) values measured by impedance spectroscopy. Averages of the last three hourly measurements before terminating the experiment (this corresponds to 40-42 h after cell seeding on filter inserts for DMM-BMECs and to 130-132 h for EECM-BMECs) are shown. Each symbol represents an independent differentiation and each symbol type corresponds to an iPSC clone (EECM-BMECs: 9 differentiations with 6 clones, DMM-BMECs: 7 differentiations with 3 clones). Measurement was done at least in triplicates per iPSC differentiation. **D)** Representative overlay histograms from flow cytometry analysis of PDGFRβ and NG2 in BPLCs at day 22 of differentiation are shown. **E)** Representative images of fluorescent staining of PDGFRβ, NG2, transgelin (SM-22), calponin and SMA (all in green) in BPLCs at day 24 of differentiation are shown. Nuclei were stained with DAPI (blue). Scale bar = 50 μm. **F)** Representative confocal images from immunofluorescent staining of occludin, claudin-3, ZO-1 and E-Cadherin (all in green) are shown. Nuclei were stained with DAPI (blue). Scale bar: 10 μm.


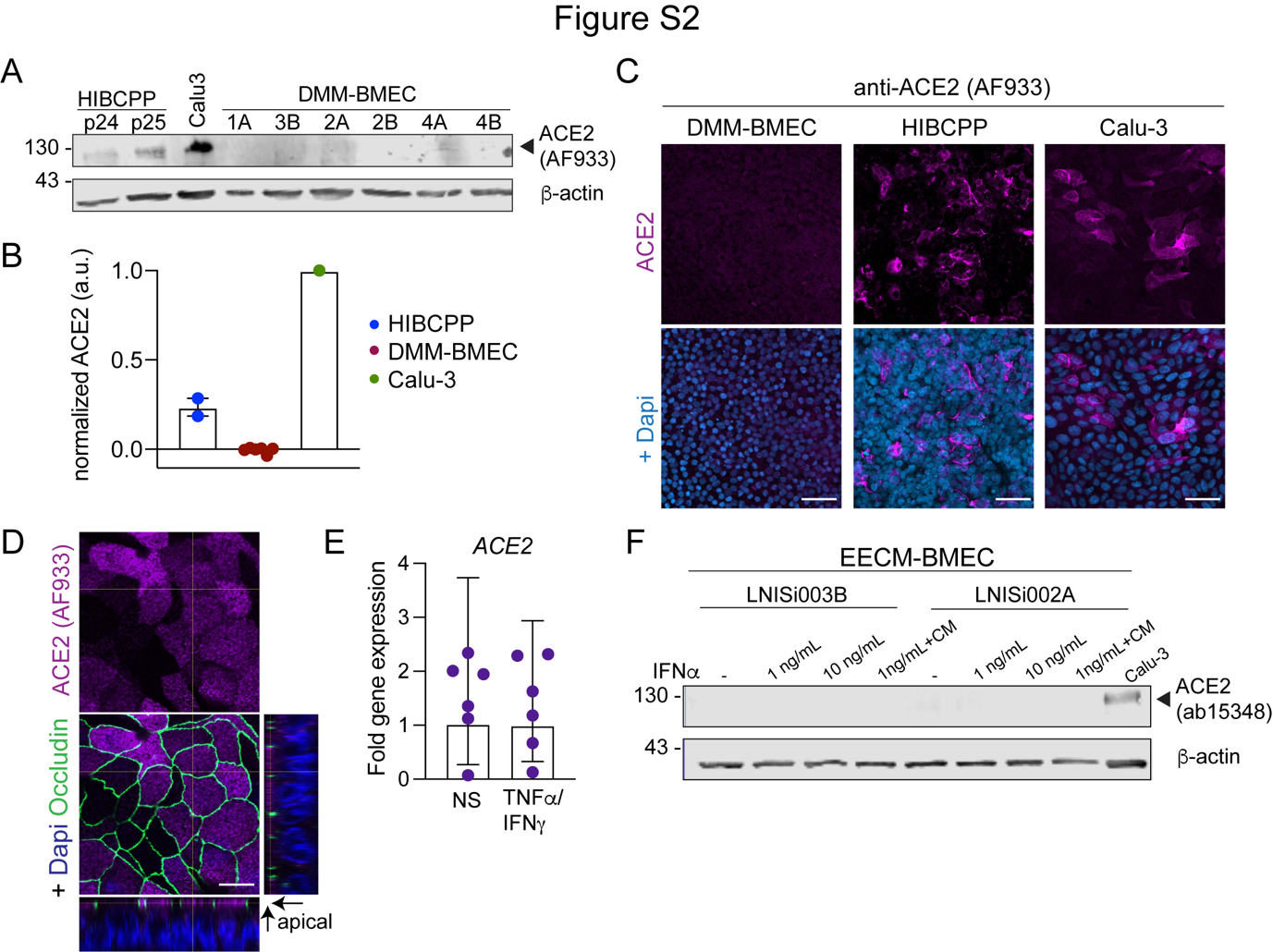


**Fig. S2. ACE2 expression in HIBCPP, DMM-BMECs and EECM-BMECs. A)** Western blot analysis of ACE2 using an antibody raised against full-length ACE2 (AF933) with β-actin as a loading control in DMM-BMECs and HIBCPP is shown. Each lane represents a differentiation of DMM-BMECs from the indicated iPSC clone and a replicate of HIBCPP with the indicated passage number. Glycosylated ACE2 is expected at 130 kDa. 25 μg protein/ lane was loaded. **B)** Quantification of relative ACE2 expression normalized to the expression in Calu-3 cells by Western blotting is shown. Each dot represents a clone for DMM-BMECs (6 iPSC clones) and a replicate of HIBCPP cells. **C)** Confocal images of immunofluorescence staining for ACE2 (AF933) in DMM-BMECs, HIBCPP and Calu-3 are shown. Nuclei were stained with DAPI (blue). Scale bar = 50 μm. The images are representative of two DMM-BMECs differentiations from two iPSC clones, and 6 independent replicates from HIBCPP and 4 from Calu-3 cells. **D)** Representative confocal close-up image of immunofluorescence staining with ACE2 (AF933) in HIBCPP as a single Z-stack and for the merge in addition orthogonal sections are shown. Scale bar = 10 μm. **E)** qRT-PCR analysis of *ACE2* in EECM-BMECs after stimulation with TNFα and IFNγ (1 ng/mL and 20 IU/mL) for 4 hours as expression fold change to non-stimulated EECM-BMECs is shown. β-actin and GAPDH were used as reference genes. Each dot represents a differentiation from a different iPSC clone. Data is shown as geometric mean with standard deviation. P-value was calculated using one-way Anova followed by Tukey’s test to correct for multiple comparison. **E)** Western blot analysis of ACE2 using ab15348 raised against the intracellular domain of ACE2 with β-actin as a loading control 24 h after treatment with 0, 1 or 10 ng/mL interferon (IFN)-α is shown. 10 μg of protein was loaded per lane. 2 iPSC-clone-derived EECM-BMECs were assessed. CM: Conditioned medium from SMLCs.

*
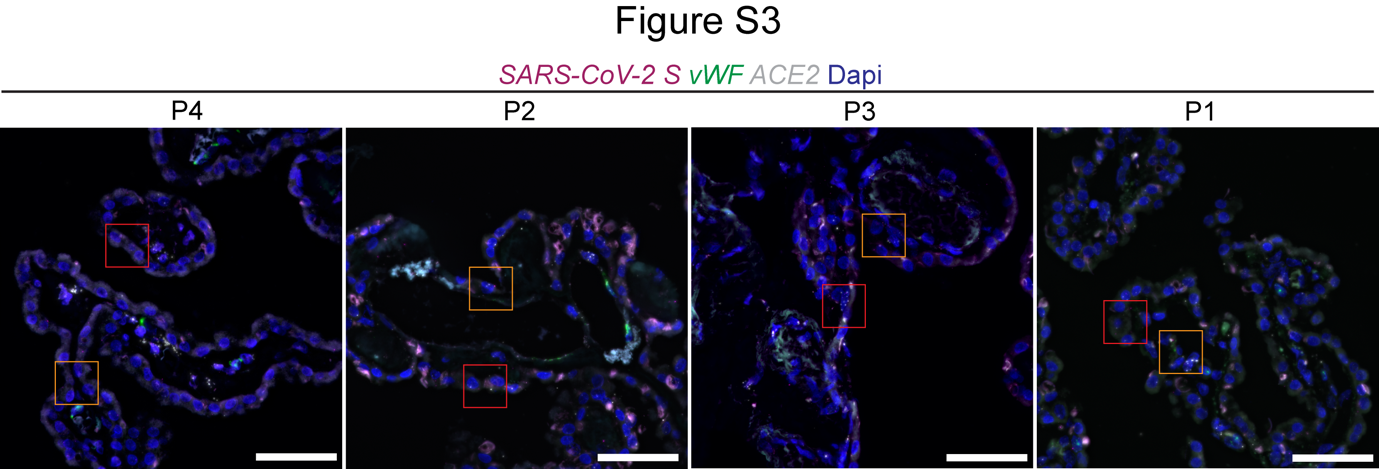
*

**Fig. S3.** **SARS-CoV-2 RNA is detected in ChP epithelial cells in COVID-19 patients.** The same representative images as in Fig. 6 of fluorescent RNA in situ hybridization for *SARS-CoV-2 S* (magenta), *vWF* (green) and *ACE2* (grey) of ChP from 4 different patients are shown without arrow heads. Nuclei were stained with DAPI (blue). All images are maximum intensity Z projections. Red and orange

boxes define area of inset. Scale bar = 50 μm.
